# Structural basis of mammalian glycan targeting by *Vibrio cholerae* cytolysin and biofilm proteins

**DOI:** 10.1371/journal.ppat.1006841

**Published:** 2018-02-12

**Authors:** Swastik De, Katherine Kaus, Shada Sinclair, Brandon C. Case, Rich Olson

**Affiliations:** Department of Molecular Biology and Biochemistry, Molecular Biophysics Program, Wesleyan University, Middletown, Connecticut, United States of America; University of California Davis School of Medicine, UNITED STATES

## Abstract

*Vibrio cholerae* is an aquatic gram-negative microbe responsible for cholera, a pandemic disease causing life-threatening diarrheal outbreaks in populations with limited access to health care. Like most pathogenic bacteria, *V*. *cholerae* secretes virulence factors to assist colonization of human hosts, several of which bind carbohydrate receptors found on cell-surfaces. Understanding how pathogenic virulence proteins specifically target host cells is important for the development of treatment strategies to fight bacterial infections. *Vibrio cholerae* cytolysin (VCC) is a secreted pore-forming toxin with a carboxy-terminal β-prism domain that targets complex *N*-glycans found on mammalian cell-surface proteins. To investigate glycan selectivity, we studied the VCC β-prism domain and two additional β-prism domains found within the *V*. *cholerae* biofilm matrix protein RbmC. We show that the two RbmC β-prism domains target a similar repertoire of complex *N*-glycan receptors as VCC and find through binding and modeling studies that a branched pentasaccharide core (GlcNAc_2_-Man_3_) represents the likely footprint interacting with these domains. To understand the structural basis of *V*. *cholerae* β-prism selectivity, we solved high-resolution crystal structures of fragments of the pentasaccharide core bound to one RbmC β-prism domain and conducted mutagenesis experiments on the VCC toxin. Our results highlight a common strategy for cell-targeting utilized by both toxin and biofilm matrix proteins in *Vibrio cholerae* and provide a structural framework for understanding the specificity for individual receptors. Our results suggest that a common strategy for disrupting carbohydrate interactions could affect multiple virulence factors produced by *V*. *cholerae*, as well as similar β-prism domains found in other vibrio pathogens.

## Author summary

Bacterial pathogens secrete multiple virulence factors to aid in infection including adhesion molecules, effector proteins, enzymes, toxins and biofilm proteins. To increase the potency and specificity of these molecules, many factors contain binding sites for host cell-surface receptors. This study involves two such factors from the human pathogen *Vibrio cholerae*: a toxin that forms cytotoxic pores in the cell-membranes of target cells (most likely immune cells like neutrophils) and biofilm matrix proteins that help form a protective sheath around growing bacterial colonies. We show that both factors utilize similar carbohydrate receptors to recognize cell surfaces. Uncovering the structural basis for how host cells are targeted is important in understanding how *V*. *cholerae* and similar organisms cause disease.

## Introduction

The recognition of carbohydrate receptors on host cell-surfaces is an important strategy for achieving the selectivity and potency of virulence factors including adhesion molecules, toxins, and biofilm proteins [1–3]. Often mediated by a canonical set of lectin domains with conserved folds, these proteins may broadly recognize terminal sugars on the end of long glycan chains, or specific polysaccharide motifs with complex branched stereochemistry [4]. Understanding the structural mechanism for glycan specificity by lectin domains is important in determining how effector proteins recognize specific host cells and for developing drugs against pathogenic proteins [5–7].

**Fig 1:**
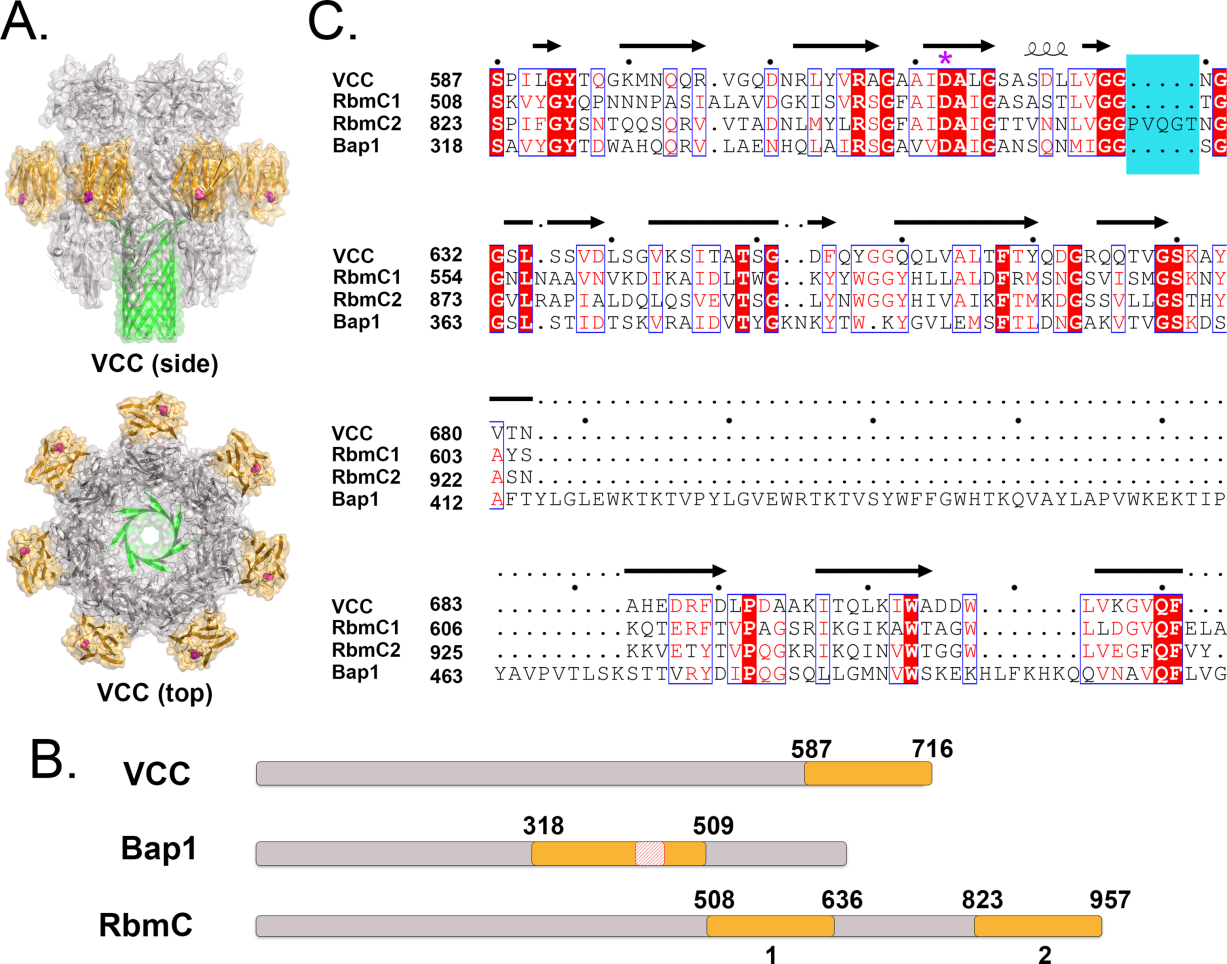
Comparison of β-prism domains from VCC, Bap1, and RbmC. (A) Crystal structure of the heptameric pore state of VCC [62] showing β-prism domains in orange and D617 (essential for carbohydrate binding) indicated by magenta spheres. (B) Schematic showing approximate locations of β-prism domains (shaded orange) in VCC, Bap1, and RbmC. Red box in Bap1 indicates the approximate location of the 58 amino acid insertion of unknown function. (C) Sequence alignment produced using MEGA7 [63] and ESPript v. 3.0 (http://espript.ibcp.fr) [64] of four *V*. *cholerae* β-prism domains. Identical residues are shaded red whereas similar residues are indicated by red type. The second RbmC β-prism domain contains a five-amino acid insertion near the glycan-binding pocket (shaded blue). VCC D617 is marked by a purple asterisk. Structure figures made using the PyMOL Molecular Graphics System, Version 1.8 (Schrödinger, LLC).

*Vibrio cholerae* is a pernicious human pathogen that secretes factors that utilize carbohydrate receptors, most notably the classical cholera toxin (CT), which binds to GM_1_ gangliosides on the intestinal epithelium [8]. *V*. *cholerae* also secretes a pore-forming toxin called *Vibrio cholerae* cytolysin (VCC), which helps defend the bacteria from the host immune system according to mouse models [9,10]. VCC recognizes complex *N*-glycans commonly found on animal cells [11] through a carboxy-terminal domain with a type I β-prism fold (Fig 1A); deletion of this domain results in a more than 99.9% loss in cytolytic activity [11]. Even though complex *N*-glycans are the preferred target of VCC, the exact footprint recognized by the VCC β-prism domain and the structural mechanism for this interaction are currently unknown.

β-prism folds in *V*. *cholerae* are not unique to VCC: three additional β-prism domains exist in two biofilm matrix proteins called RbmC (*r*ugosity and *b*iofilm structure *m*odulator *C*) [12] and Bap1 (*b*iofilm *a*ssociated *p*rotein 1) [13] (Fig 1B). The formation of biofilms by *V*. *cholerae* is an important survival strategy [14–16] that facilitates bacterial attachment to surfaces, helps protect against environmental insults [17], and is also implicated in human transmission of the disease [18,19]. The biofilm itself is primarily composed of secreted molecules including an exopolysaccharide [17] called VPS (*V*ibrio *p*oly*s*accharide) assembled and exported by two clusters of VPS-related genes (*vps I* and *vps II*) [20], several matrix proteins produced by the *rbm* gene cluster, and a mixture of nucleic acids and small biomolecules [20]. RbmC and Bap1 are related multidomain proteins with overlapping functions [12,21], involved in the surface attachment of biofilms and encapsulation of growing cell clusters [21–23]. Simultaneous deletion of RbmC and Bap1 leads to colonies deficient in biofilm formation, however, either gene is sufficient to restore function [21]. Interestingly, mass spectral analysis of isolated biofilm material shows that RbmC, Bap1, and VCC are all retained in the biofilm matrix and may therefore display affinity for a subset of related ligands [21].

To better understand the structural specificity of β-prism-carbohydrate interactions in *V*. *cholerae* biofilm proteins and toxins, we cloned and expressed the two β-prism domains from RbmC and determined their glycan specificity by screening against a chip-based mammalian glycan library [24,25]. Using isothermal titration calorimetry (ITC) and a fluorescence-based binding assay, we determined the binding affinity of the vibrio β-prisms to multiple fragments of target *N*-glycans to determine the unique footprint recognized by these domains. We crystallized and solved the structure of one β-prism domain from RbmC bound to two fragments of the glycan core. Finally, we show that VCC and both RbmC β-prism domains can target glycans present on rabbit blood cells, a model system that contains complex *N*-glycans on the cell surface. Our results illustrate a common structural mechanism by which vibrio toxin and biofilm β-prism domains target glycans on host cell-surfaces, facilitating cell lysis in one case and cell attachment in another. Our structures provide a model for how *Vibrio cholerae* targets the invariant core of cell-surface receptors commonly found on vertebrate cells while allowing for heterogeneity in the rest of the glycan, a model that may apply to β-prism domains found elsewhere in nature.

## Results

### The *V*. *cholerae* genome contains four β-prism domains

A protein BLAST [26] search (blastp) was conducted using the C-terminal *Vibrio cholerae* cytolysin β-prism domain (residues 587 to 716 from PDB ID 1XEZ in *Vibrio cholerae* strain N16961), which identified three additional β-prism domains in two open reading frames (Fig 1B), belonging to *rbmc* (NCBI gene ID = 2614150) and *bap1* (NCBI gene ID = 2613517), which encode biofilm matrix proteins [12,27]. For simplicity, we refer to the two β-prism domains of RbmC as RbmC1 and RbmC2 (Fig 1B). A sequence alignment of the four *V*. *cholerae* β-prism domains (Fig 1C) indicates that the degree of sequence identity between VCC and the other three domains is 36.5%, 40.5%, and 33.3%, for RbmC1, RbmC2, and Bap1, respectively. Attempts at expressing full-length RbmC and Bap1 proteins in *E*. *coli* were unsuccessful, however expression of the two β-prism domains from RbmC was achieved via generation of a fusion construct with bacterially optimized GFP_UV_. Unfortunately, expression of the Bap1 β-prism domain in a soluble form was not possible, even as a GFP_UV_-fusion construct.

### Glycan-binding repertoire of RbmC β-prism domains

**Fig 2:**
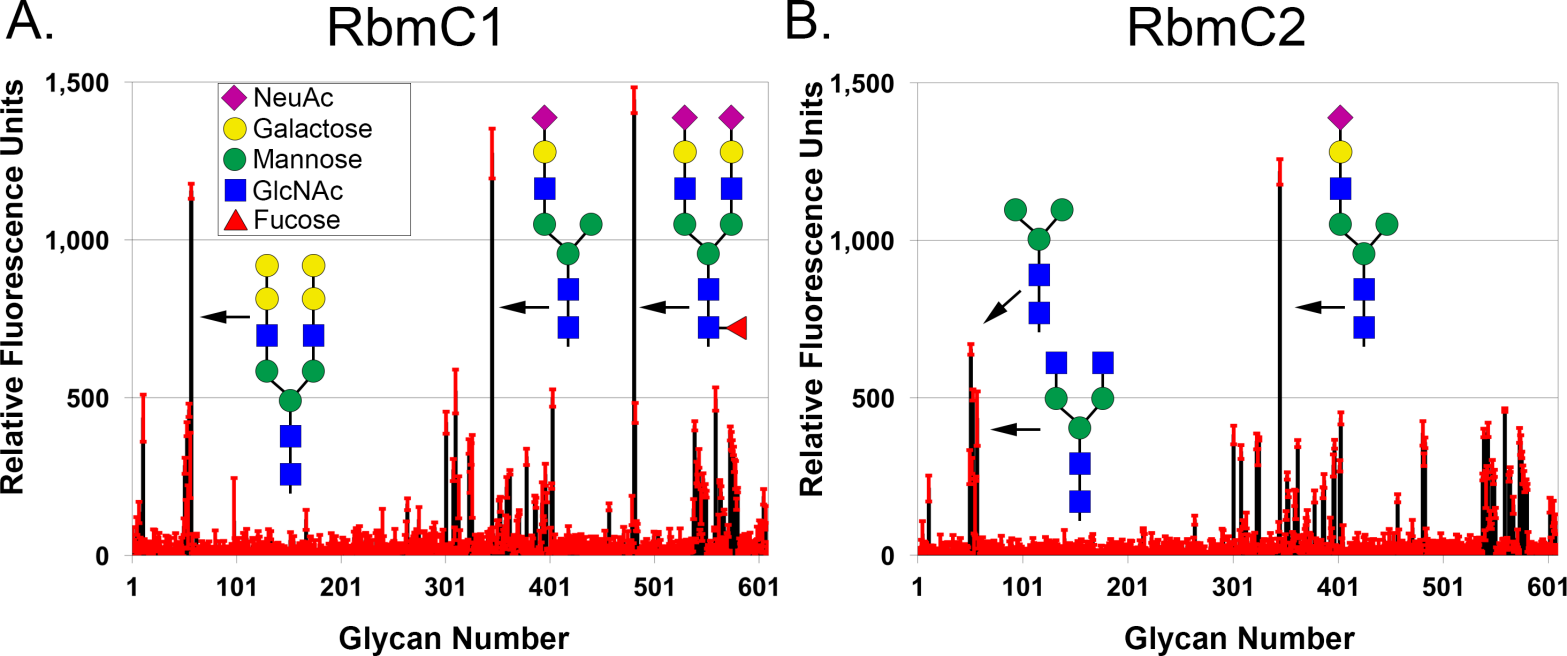
Glycan chip results for RbmC1 and RbmC2. Glycan chip data is represented in relative fluorescence units with the standard deviation of four replicates indicated by red error bars for (A) RbmC1 and (B) RbmC2. The top glycan hits are shown in schematic representation based on the key (inset). Fragments of complex *N*-linked glycans make up most positive hits.

To determine the glycan specificity of RbmC1 and RbmC2, we labeled the purified proteins with a fluorescent tag and subjected them to glycan screening by the Consortium for Functional Glycomics (CFG) against the mammalian glycan chip v. 5.2, which contains 609 mammalian glycans arrayed via amino linkers on an *N*-hydroxysuccinimide-activated glass slide (http://www.functionalglycomics.org/). Screening results indicated a similar pattern of glycan recognition between RbmC1, RbmC2, and VCC [11], with top hits containing a similar pattern of biantennary complex *N*-glycans (Fig 2A and 2B). The glycans identified in the screen typically contained an NGA2-type conserved heptasaccharide motif (GlcNAcβ1-2Manα1-6(GlcNAcβ1-2Manα1–3)Manβ1-4GlcNAcβ1-4GlcNAc), although binding to some glycans missing one or both antennae (truncated at the mannotriose core, see Fig 2) was also observed for all three proteins. The top four previously determined VCC hits [11] all contained a complete heptasaccharide NGA2 core, although there was one single-antenna complex *N*-glycan within the top 10 glycans identified. As was observed for VCC, binding to high-mannose type glycans (which contain highly-branched mannose chains past the mannotriose core) was not observed. The glycan chip results are not necessarily quantitative, meaning the fluorescence signal should not be interpreted as being directly proportional to the binding affinity since the glycan density on the chip may vary. For this reason, further binding experiments were required to determine the affinity of the target proteins for individual glycan fragments.

Our results show that the VCC toxin and RbmC β-prisms target a similar repertoire of carbohydrate moieties on cell surfaces, suggesting a common strategy of cell-surface recognition. While complex *N*-linked glycans are found in all higher eukaryotes including plants and animals [28], later processing steps in the Golgi vary across different organisms yielding differently modified repertoires of cell-surface glycans [29,30]. Complex *N-*glycans are abundantly expressed on differentiated epithelial cells in the gastrointestinal tract [31] and are also present on mucin proteins [32]. While the processing of N-glycans on surface proteins yield a heterogeneous mixture of branching and modification characteristics, the core structures found in the screen are likely present in glycans found on the intestinal epithelium.

Owing to the design of the glycan chip, our results indicate that the β-prism domains of VCC and RbmC are capable of targeting mammalian cell-surface glycans. While it is possible that the β-prism domains of RbmC might also bind to the exopolysaccharide abundant in *V*. *cholerae* biofilms (VPS), it is reasonable to posit that binding of this tetrasaccharide repeat is preferentially coordinated by any of the additional domains found in RbmC, or by other biofilm matrix proteins, and that the primary role of the RbmC β-prism domains is to target host cell glycans. This hypothesis is supported by the strong affinity (low nanomolar) of VCC and RbmC β-prisms for *N*-glycans and absence of hits that resemble the VPS tetrasaccharide repeat [33]. Additionally, another biofilm matrix protein, RbmA, has been implicated in VPS binding in the *V*. *cholerae* biofilm [34].

### Mapping glycan binding by vibrio β-prism domains

**Fig 3:**
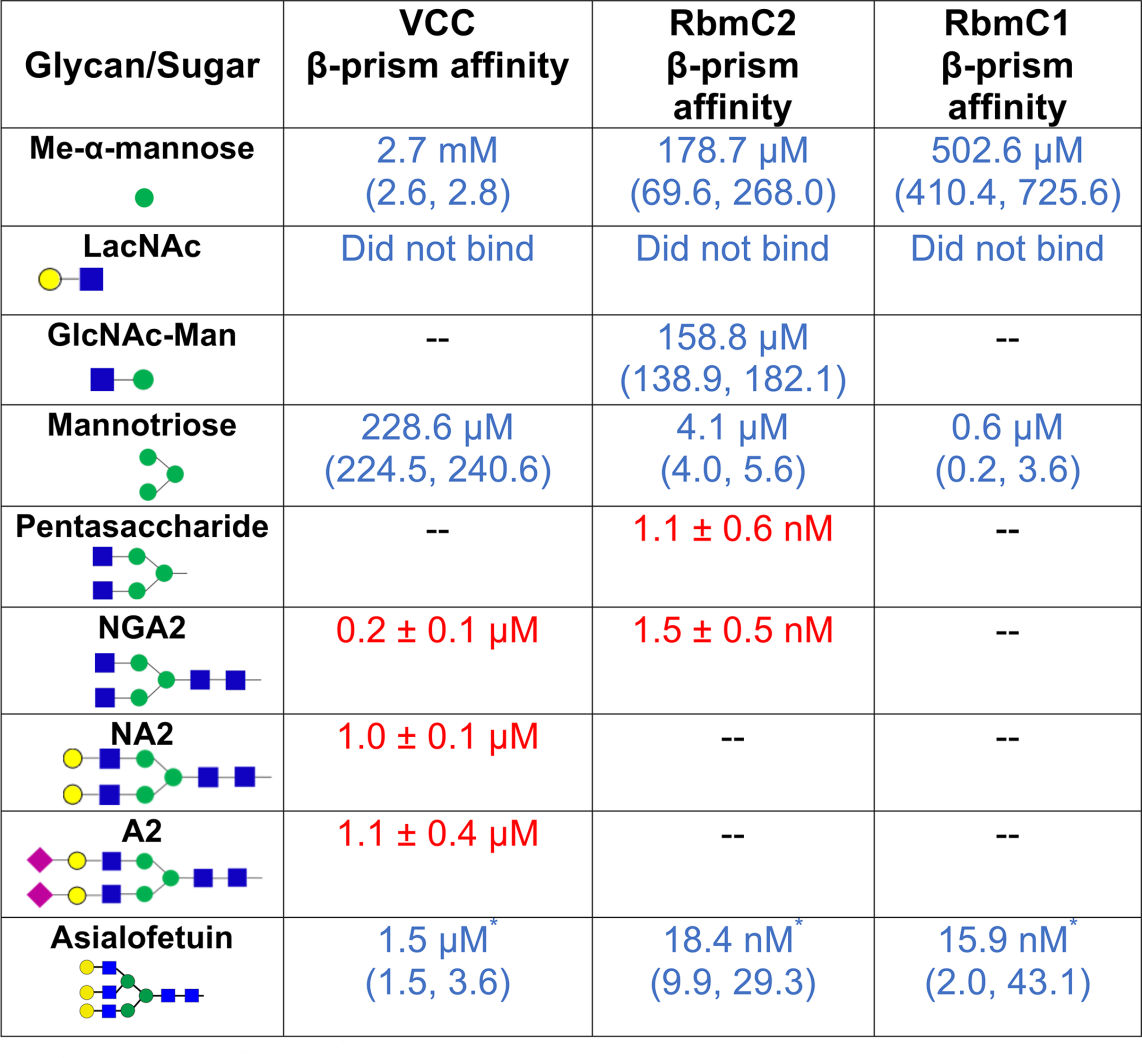
Glycan binding affinities. Binding affinities for various glycans were determined using ITC (blue) or intrinsic tryptophan fluorescence spectroscopy (red). Errors are reported as 95% confidence levels (for ITC data) or the standard error of the mean (for fluorescence data). Dashes represent experiments not carried out due to lack of feasibility. Carbohydrates are represented as described in Fig 2A. *Asialofetuin is a glycoprotein containing three primary glycosylation sites, which contain a mixture of bi- and tri-antennary glycans [35]. Data were fit assuming three sites per glycoprotein, but due to the heterogeneity of glycosylation these numbers may represent an overestimation of the binding affinity. The asialofetuin schematic shows the predominant tri-antennary glycan.

To better understand of the binding footprint for complex *N*-glycan recognition by vibrio β-prism domains, we measured the binding affinity of the VCC and RbmC β-prisms to complex *N*-glycan fragments using a top-down approach (Fig 3 and S1 Fig). Due to the variable availability of different glycan fragments, both isothermal titration calorimetry (ITC) and intrinsic tryptophan fluorescence spectroscopy were utilized in characterizing the glycan footprint of the vibrio β-prism domains. For glycan fragments that could not feasibly be obtained in the quantities required for ITC measurements, the intrinsic tryptophan fluorescence of purified β-prism domains was used to monitor ligand association.

The footprinting results indicate that the VCC β-prism binds most tightly to the NGA2 heptasaccharide core, with a measured binding affinity of 0.2 μM. Glycan fragments smaller than NGA2 bound less tightly, including a 228.6 μM affinity for the mannotriose branch point and 2.7 mM for the methyl-α-mannose monosaccharide (as reported previously in [11]). We were unable to detect binding for the *N*-acetyl-D-lactosamine (LacNAc) disaccharide, which is present in one arm of a typical complex *N*-glycan. Glycans with arms longer than NGA2, including the NA2 and A2 glycans (which respectively extend galactose and sialic acid modifications onto the terminal ends of the biantennary arms), did not display tighter binding affinities. To investigate whether the vibrio β-prism domains are also capable of binding a mammalian-derived glycoprotein, we measured the binding affinity of VCC to bovine asialofetuin. While asialofetuin is a good indicator of general binding to complex *N*-glycans, analysis of these binding phenomena are overestimated by ITC because the glycoprotein contains three glycosylation sites occupied by heterogeneous mixtures of bi- and tri-antennary glycans [35]. VCC bound this protein as previously reported [36] with an affinity in the low micromolar range.

Binding studies were repeated for several of the glycan fragments with the RbmC1 and RbmC2 β-prism domains. In contrast to the 2.7 mM methyl-α-mannose affinity with VCC, RbmC1 and RbmC2 displayed tighter affinities of 178.8 μM and 502.6 μM, respectively (Fig 3). We were also unable to observe binding of RbmC1 or RbmC2 to the LacNAc disaccharide, while RbmC2 did bind *N*-acetylglucosaminyl-β-1,2-mannose (GlcNAc-Man) with an affinity of 158.8 μM. The mannotriose core (1,3-α-1,6-α-D-mannotriose) also bound to the RbmC1 and RbmC2 β-prism domains with affinities of 0.6 μM and 4.1μM, respectively. Binding of NGA2 to RbmC2 exhibited one of the highest affinities measured for any β-prism domain to a glycan fragment, with an affinity of 1.1 nM. A pentasaccharide fragment of NGA2 missing the two anchoring GlcNAc sugars provided a similar affinity of 1.5 nM, which is still much tighter than binding to mannotriose (4.1 μM). This suggests that at least for RbmC2, it is the pentasaccharide core that is the likely footprint targeted by the β-prism domain. RbmC1 and RbmC2 also bound tightly to asialofetuin, with apparent affinities of 15.9 nM and 18.4 nM, respectively.

If we assume that the three β-prism domains employ a similar footprint for glycan recognition (as supported by the glycan chip data), our results indicate that the binding footprint is likely centered around the mannotriose core. At least for VCC, glycans larger than NGA2 do not bind any tighter, likely because the extended chains do not strongly interact with the β-prism. Similarly, for RbmC2 the pentasaccharide and NGA2 glycans had similar affinities (but better than mannotriose) and asialofetuin did not bind any better than the pentasaccharide. It is possible that the minimal recognition motif is comprised of a tetrasaccharide made up of mannotriose plus a single *N*-acetylglucosamine, however due to the difficulty in obtaining asymmetrically branched glycans, this interaction could not be tested. High-mannose type glycans (or oligomannose, containing only mannose past the mannotriose core) are present on the mammalian glycan chip, but did not elicit strong binding by any of the vibrio β-prism domains tested. In general, the RbmC β-prism domains bound more tightly to glycan fragments than the VCC toxin domain by a factor of approximately 50-100-fold.

### Structure of the RbmC2 β-prism domain bound to mannotriose

**Fig 4:**
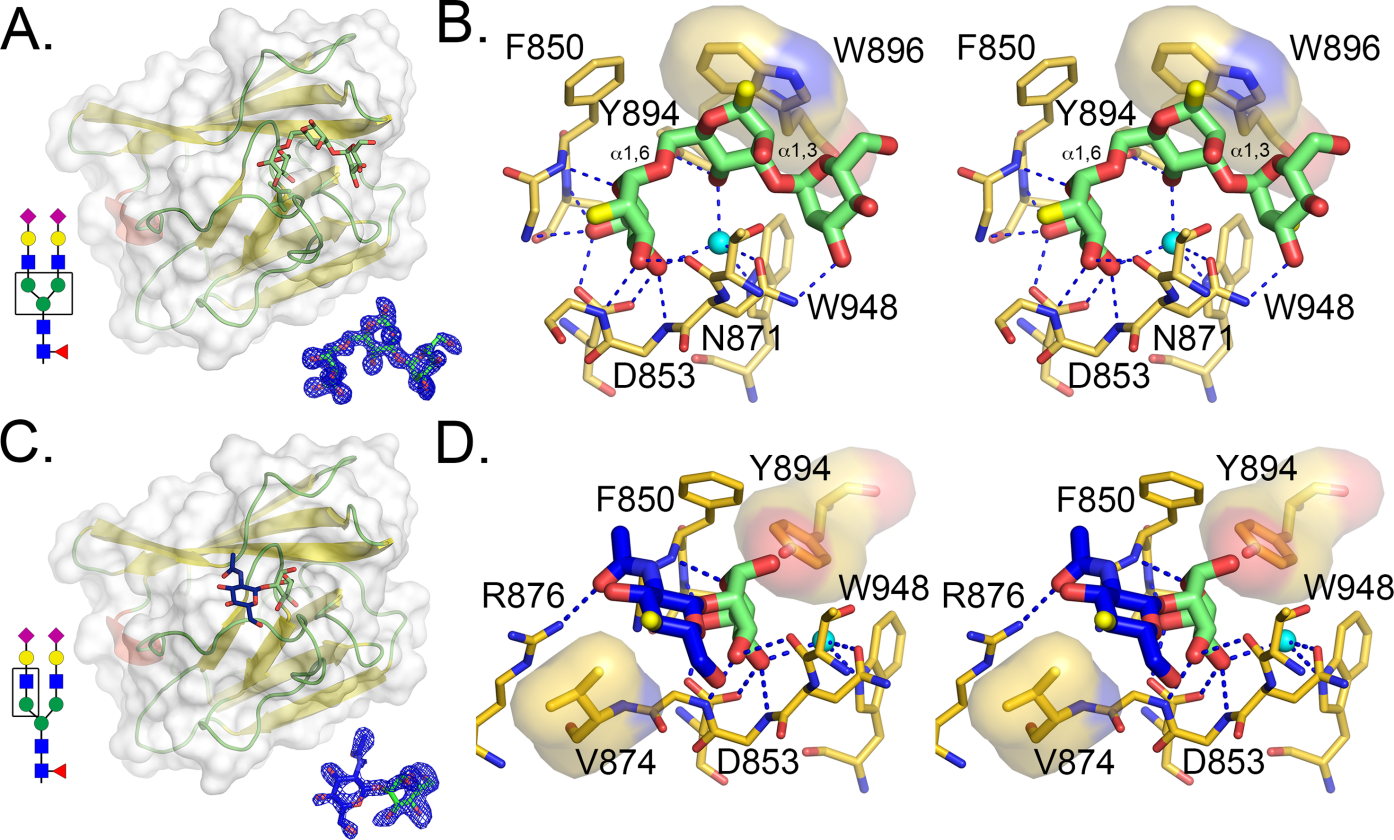
Crystal structures of RbmC2 with mannotriose and GlcNAc-Man bound. (A) Crystal structure of the isolated RbmC2 β-prism domain with mannotriose (1,3-α-1,6-α-D-mannotriose) shown in stick representation. The mannotriose core in a typical complex *N*-glycan is boxed (lower left, see key in Fig 2A). A simulated annealing OMIT electron density map contoured to 2.5 σ for the ligand is displayed in blue mesh, duplicated and extracted from the overall structure for clarity (lower right). (B) Stereo view of residues that directly interact with the mannotriose ligand. Putative hydrogen bonds are displayed as blue dotted lines and van der Waals contacts are denoted with a semi-transparent surface representation. Within the mannotriose ligand (green sticks), the attachment point of connecting saccharides in an intact complex *N*-glycan are colored yellow. A water molecule (B-factor = 4.18 Å^2^) (blue sphere) is held in a tetrahedral coordination sphere involving N871, W948, and two hydroxyl groups from the mannotriose glycan fragment. (C) Same as in A, but showing the GlcNAc-Man-bound structure, solved to 1.8 Å resolution. (D) Same as in B, but for the GlcNAc-Man structure.

To gain insight into to the mechanism of glycan recognition by vibrio β-prism domains, we performed crystallization trials in the presence of *N*-glycan core fragments. Although we were unable to obtain well-diffracting crystals of any β-prism domain with a bound pentasaccharide fragment, crystals were obtained for RbmC2 bound to mannotriose and *N*-acetylglucosaminyl-β-1,2-mannose (GlcNAc-Man). X-ray data were collected to 1.5 Å resolution and the structure solved by molecular replacement using the VCC β-prism lectin domain (Table 1). The electron density map displayed unambiguous density for the mannotriose ligand in a single orientation (Fig 4A). Calculations using PISA [37] indicated 384 Å^2^ of buried surface area between the ligand and the protein. As previously observed in VCC and other β-prism lectins, a conserved aspartic acid residue (D617 in VCC, D853 in RbmC2) mediates hydrogen-bonding interactions with hydroxyl groups on one of the mannose rings (Fig 4B). In VCC, mutation of this residue to alanine results in a 50-fold loss in hemolytic activity of the toxin. In the RbmC2 structure, D853 interacts with the α1,6-linked mannose, whose O4 and O6 hydroxyl groups make putative hydrogen-bonding interactions with the D853 side-chain. This position is highly conserved across β-prism domains found throughout nature and is likewise found in all four of the vibrio β-prism domains expressed by *V*. *cholerae* (Fig 1C).

**Table 1:** X-ray and refinement statistics.

	RbmC2 lectin- Apo	RbmC2 lectin- Mannotriose	RbmC2 lectin- GlcNAc(β1–2)Man
**Data collection**			
Space group	C222_1_	P2_1_	P2_1_
Cell dimensions *a*,*b*,*c* (Å)	90.6, 92.7, 100.3	31.9, 38.6, 51.0	31.9, 38.8, 51.9
Cell angles *α*,*β*,*γ* (°)	90, 90, 90	90, 101.9, 90	90, 105.3, 90
Resolution (Å)	16.0–2.2 (2.27–2.20)*	16.1–1.5 (1.58–1.50)	38.8–1.8 (1.90–1.80)
*R*_*sym*_	0.35 (1.4)	0.08 (0.48)	0.08 (0.39)
*R*_*pim*_	0.14 (0.57)	0.06 (0.43)	0.05 (0.30)
*I/σI*	7.5 (2.1)	7.7 (1.5)	9.6 (2.1)
Completeness (%)	99.7 (100.0)	95.1 (86.1)	91.9 (56.0)
Redundancy	7.1 (6.7)	2.6 (1.8)	3.9 (2.6)
*CC*_*1/2*_	0.978 (0.624)	0.995 (0.580)	0.996 (0.704)
Wilson B-factor (Å^2^)	5.2	3.1	7.7
**Refinement**			
Resolution (Å)	16.5–2.2	15.9–1.5	24.1–1.8
No. of reflections	21,727	18,617	10,565
*R*_*work*_*/R*_*free*_ (%)	17.3/21.7	16.4/19.2	16.6/18.9
Protein molecules/asymmetric unit	3	1	1
**No. of atoms**			
Protein	3,056	1,042	1,038
Ligand	36	76	64
Water	477	237	215
**R.M.S. deviations**			
Bond lengths (Å)	0.003	0.004	0.003
Bond angles (°)	0.6	0.7	0.6
**Ramachandran statistics**			
Favored	98.2%	97.8%	97.8%
Allowed	1.8%	2.2%	2.2%
Outliers	0.0%	0.0%	0.0%
**MolProbity Scores**			
Overall score	0.9 (100%)	1.1 (99%)	1.1 (100%)
All-atom clashscore	1.6 (100%)	2.8 (98%)	2.8 (99%)

*Numbers in parentheses denote the highest resolution shell. MolProbity percentiles in parentheses are for structures at a similar resolution [38].

Analysis of bonding interactions within a distance less than 3.35 Å indicated a total of ten direct hydrogen bonds between RbmC2 and the mannotriose ligand (Figs 4B, S2 and S3), with eight of these interactions targeting the α1,6-linked mannose. Of the eight hydrogen bonds with the α1,6-linked mannose, six are interactions with the peptide backbone, and two are with the oxygens of the D853 side-chain. The position occupied by the α1,6-linked mannose is the same as that observed in the structure of the VCC β-prism in complex with the monosaccharide, methyl-α-mannose [11]. The α1,6-linked mannose also makes the most extensive surface contacts with RbmC2, with 165 Å^2^ of buried surface area. The central and α1,3-linked mannose rings each make a single hydrogen bond to the protein, both to side-chain atoms. Not surprisingly, the two more “loosely”-bound sugars had higher average crystallographic B-factors (3.5 Å^2^, 7.4 Å^2^, and 14.0 Å^2^ for the α1,6-linked, central, and α1,3-linked mannose sugars, respectively). Binding of RbmC2 to these saccharides also resulted in progressively less buried surface area at 121 Å^2^ and 98 Å^2^ for the central and α1,3-linked mannose, respectively. The high resolution of the structure confirmed that the central mannose best refined as the β-anomer, as expected [30]. Also of note is a centrally located water molecule (B-factor = 4.2 Å^2^) coordinated between ligand and protein groups (Figs 4B and S3), including W948 and N971. The latter residue is on a flexible loop that adopts a different conformation in the apo structure, suggesting that this particular water molecule is only present when the glycan is bound.

### Structure of the RbmC2 β-prism domain bound to GlcNAc-Man

RbmC2 was crystallized in the presence of *N*-acetylglucosaminyl-β-1,2-mannose (GlcNAc-Man), and the structure solved to 1.8 Å resolution. Density for the GlcNAc-Man ligand was observed in the same binding pocket identified in the RbmC2/mannotriose and VCC β-prism/methyl-α-mannose complexes. While occupancy refinement of the mannotriose sugar suggested near full occupancy, the *N*-acetylglucosaminyl-β-1,2-mannose ligand density suggested a mixture of the carbohydrate and glycerol (overlapping with the C4, C5, and C6 carbons of the mannose sugar). Alternate configurations for the two ligands were built into the density and their relative occupancies were refined, yielding a ratio of 65% GlcNAc-Man to 35% glycerol (presumably from the cryoprotectant). The mannose moiety occupies the same location as the α1,6-linked mannose in the mannotriose structure (Fig 4C), making a similar constellation of hydrogen-bonding interactions. Coordination of the disaccharide by RbmC2 resulted in a total of 338 Å^2^ of buried surface area with a contribution of 160 Å^2^ by the mannose moiety and 179 Å^2^ by the *N*-acetylglucosamine. Interestingly, the centrally located water molecule observed in the mannotriose structure was also present, even though the fourth coordination site (previously made by the central mannose moiety) is missing in this structure (Figs 4D and S4). In the apo structure (with three protein molecules in the asymmetric unit), glycerol molecules from the cryo-protection solution are found in the glycan binding pocket. In two copies of the asymmetric unit, a water is present bound to W948 as in the mannotriose and GlcNAc-Man structures. In the third copy, a hydroxide group from a glycerol molecule replaces this position. As previously stated, the loop containing the N871 residue is unstructured in two of three copies of the asymmetric unit and the ordered copy is in a different conformation than in the ligand-occupied structures. This suggests that binding of the ligand may lead to the ordering of this loop and that the water molecule coordinated by N871 and W948 may help mediate this interaction.

The *N*-acetylglucosamine moiety of the GlcNAc-Man structure was observed projecting away from the mannotriose binding cleft. Fewer hydrogen-bonding contacts are made between RbmC2 and this sugar, perhaps contributing to the higher B-factors and less well-defined electron density for this group. In fact, only a single hydrogen bond was observed with the GlcNAc group: between the acetyl O7 atom and the guanidinium group of R876. This residue is not conserved among the various vibrio β-prism domains, but is instead replaced by an asparagine in the RbmC1 sequence (and presumably structure), while in the VCC β-prism structure this location is occupied by a serine, which is also present in the Bap1 β-prism sequence. The only other observed contact between RbmC2 and the GlcNAc moiety is a van der Waals contact between the sugar ring and the side-chain of V874. The distance between the CG2 carbon of this valine and the C4 carbon of GlcNAc is 3.6 Å. This residue is not conserved across the other three β-prism domains, suggesting that interactions with this sugar may vary between the different vibrio β-prism domains.

### RbmC2 β-prism domain apo structure

RbmC2 apo crystals were obtained with space group C222_1_, with three copies in the asymmetric unit. In general, RbmC2 only displayed slight differences between the apo, mannotriose, and GlcNAc-Man structures. The all-atom RMSD between the two ligand-bound structures is 0.21 Å^2^ and approximately 0.34 Å^2^ between liganded and apo (compared to 1.2 Å^2^ for RbmC2 vs. VCC β-prism). Comparing glycan-bound structures to the three copies of RbmC2 in the apo structure asymmetric unit revealed differences in a loop (with the sequence PVQGT), which is not present in the other vibrio lectin domains (Figs 1C and S5). Threonine 870 is the only amino acid within this loop that directly contacts the ligand forming a hydrogen bond between the peptide backbone and O3 of the α1,6-linked mannose group. Aside from the rearrangement of this loop, only subtle side-chain rearrangements of F850, W896, and W948 are observed between the apo and mannotriose-bound, and GlcNAc-Man-bound structures.

Taking all of the observed protein-ligand interactions into account, the structural basis for the increased binding affinity of the RbmC2 domain over VCC is likely due to substitutions of several key residues that interact with the bound ligand. The F850 and W896 residues that participate in van der Waals and ring-stacking interactions with the mannotriose ligand are conserved in RbmC1 and RbmC2, but replaced by an alanine (A614) and tyrosine (Y654), respectively, in VCC. The increased surface area available for ring-stacking due to the substitution of tryptophan in RbmC1 and RbmC2 for the tyrosine in VCC may account for some of the increased affinity of the RbmC domains for glycans. Furthermore, the side-chain hydroxyl group of Y894 that forms a hydrogen bond with the central mannose group in the RbmC2/mannotriose structure is conserved in RbmC1, but replaced with a hydroxyl-lacking phenylalanine in VCC. The remaining residues contacting the ligand through side-chain or backbone interactions are either conserved in all three domains, or between VCC and one of the two RbmC β-prism domains.

### Effects of mutations on vibrio β-prism function

**Fig 5:**
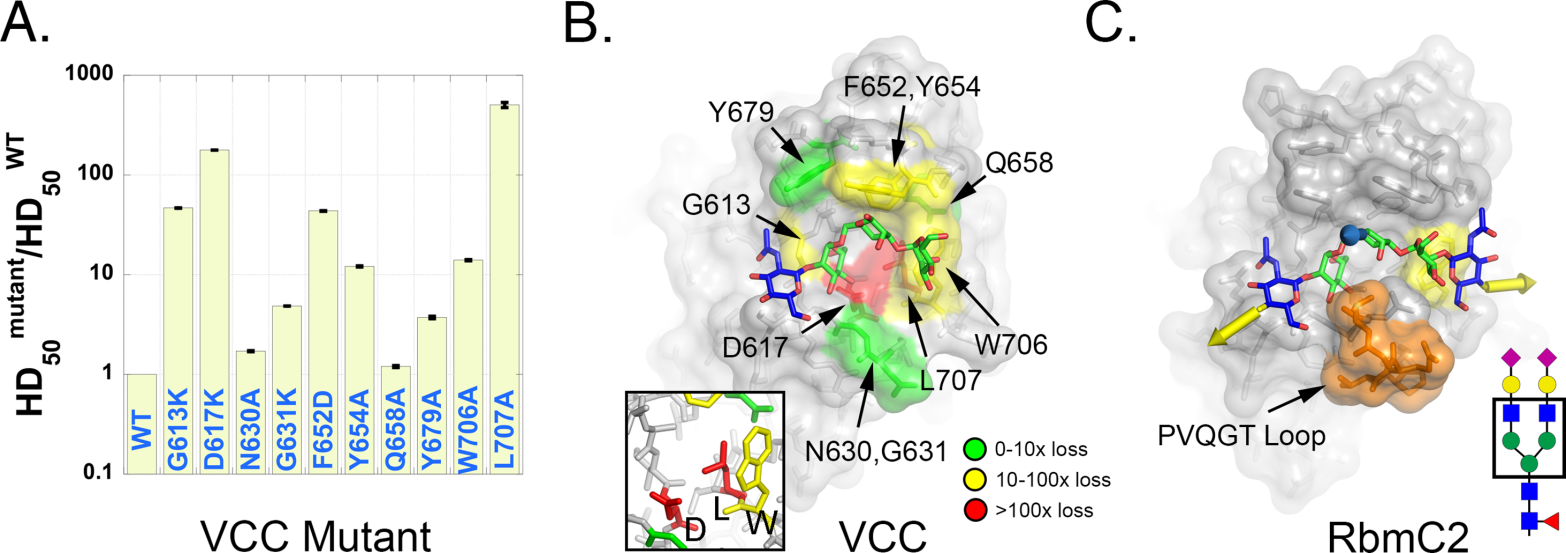
Structure/function implications of glycan binding. (A) The effect of selected mutations on the hemolytic activity of VCC is displayed as the ratio of the concentration of half-activity (HD_50_) for each mutant relative to wild-type toxin. (B) Crystal structure of the VCC β-prism domain with the composite GlcNAcMan_3_ model superimposed. The effects of mutations described in (A) are color coded and displayed on the protein surface. The inset box shows a closeup of the D617, L707, W706 core in a similar orientation. (C) Model for complex *N*-glycan binding based on the RbmC2 mannotriose structure, GlcNAc-Man structure, and mutagenesis data. The pentasaccharide core is shown boxed on a schematic representation of a complex *N*-glycan. Yellow arrows indicate the point of attachment of the subsequent two galactosyl moieties in a typical glycan and blue arrow denotes the attachment point of the double N-acetylglucosamine stem that attaches the glycan to asparagine residues on cell-surface proteins. The RbmC2 PVQGT loop insertion is colored orange and the surface of W706 colored yellow.

To gain a better understanding of the functional importance of complex *N*-glycan interactions, we made mutations to residues that line the ligand binding pocket. Because we do not have a functional assay for RbmC biofilm activity, mutations were made in VCC, where the lytic activity against mammalian cells (rabbit erythrocytes in our model system) can be monitored. As an enteric pathogen, *Vibrio cholerae* is unlikely to encounter erythrocytes during an infection. However, we demonstrated previously that VCC lyses rabbit blood cells as well as human T-cells, monocytes, and neutrophils [36], the latter of which is a likely target [10]. Furthermore, rabbit erythrocytes are highly decorated with biantennary complex *N*-glycans [39] making them a good model for targeting studies. The change in activity of the mutant is reported as the ratio of HD_50_ values (the concentration that elicits 50% cell lysis, Fig 5A) between the mutant and wild-type toxin (which is typically around 100 pM [36]).

We classified the effects of individual mutations (Fig 5B) into those that elicited a 0-10-fold loss (10–100% WT activity), 10-100-fold loss (1–10% WT activity), and >100-fold loss in activity (<1% WT activity). To put these data into context, we built a composite ligand model in which we extended the mannotriose core outwards, including the next two β1,2-linked *N*-acetylglucosamine moieties seen in complex *N*-glycans (Fig 5C). We used the GlcNAc-Man structure to place the GlcNAc moiety connected to the α1,6-linked mannose, and modeled in the second GlcNAc moiety, followed by energy-minimization. D617K, a mutation targeting the residue that forms two hydrogen bonds with the α1,6-linked mannose group in both VCC and RbmC2 (S2 Fig), elicited a 178-fold loss in activity. A previous study showed that D617K and D617A had similar deleterious effects on VCC activity [11]. Not expected was that mutation of L707 to alanine, a residue that forms the floor of the binding pocket and is conserved between three of the four vibrio β-prism domains (S2 Fig), led to a 505-fold loss in activity. Mutations resulting in 10 to 100-fold activity loss included three aromatic residues that contact the ligand in RbmC2: F652, Y654, and W706 (VCC numbering). W706 adopts two distinct rotamer conformations across several crystal structures of VCC [11] and could potentially form a stacking interaction with the *N*-acetylglucosamine residue attached to the α1,6-linked mannose (yellow in Fig 5C). To see how disrupting the *N*-acetylglucosamine residue that exits the opposite side of the binding pocket (attached to the α1,3-linked mannose) might affect binding, we mutated G613 (a glycine in all three lectin domains investigated here) to a lysine residue to sterically block the glycan exit channel. Lysine was selected as it is a bulky side-chain that is not tightly constrained in rotamer positions with a positive charge to maintain protein solubility. This mutation led to a 47-fold loss in activity suggesting that this side of the binding channel must remain unoccluded to allow the glycan arm to exit the pocket (Fig 5C). While mutation of glycine residues to any amino acid can cause issues with allowed backbone Ramachandran angles, the two glycine mutants could still be expressed in a soluble form and retained some hemolytic activity, suggesting that the mutations did not lead to a substantial misfolding of the domain.

Several mutations only displayed modest effects on the activity of the VCC toxin. N630 and G631 line the bottom lip of the binding pocket in a similar position to the attachment point of the PVQGT loop in RbmC2. The mutants N630A and G631K led to 1.7-fold and 4.9-fold losses in activity, respectively, indicating that these loop side-chains do not interact substantially with bound glycans in VCC. Likewise, Q658A had virtually no effect (1.2-fold loss) on VCC activity. This residue is at a position just outside W706 designating the outer boundary of the glycan footprint extending from the α1,3-linked mannose. Together, these results support a model where the bound glycan projects antennae outward through both sides of the binding pocket (Fig 5C). Such a bound conformation would tolerate both heterogeneity in the *N*-glycan branches, as well as core fucosylation of the first *N*-acetylglucosamine that attaches the glycan to cell-surface proteins (a common modification on glycosylated proteins [30]).

### Binding of RbmC2 to mammalian cells

To determine whether the RbmC1 and RbmC2 β-prism domains can recognize mammalian cells with complex *N*-glycans on their surface, we used GFP_UV_ fusion proteins to label rabbit whole blood cells and imaged with fluorescence microscopy. Attempts to label cells with a GFP_UV_-VCC were unsuccessful, possibly due to the lower affinity of VCC for complex *N*-glycans. To ensure that our imaging assay reflected specific binding to these cells, we made the analogous point mutation in RbmC1 (D539A) and RbmC2 (D853A) to the D617 mutation in VCC that greatly diminishes hemolytic activity against rabbit cells. ITC experiments against bovine asialofetuin confirmed that the wild-type fusion was active (S6 Fig) and that the mutant fusion exhibited greatly diminished binding. ITC experiments also identified a lack of binding of RbmC2 D853A to mannotriose. Both constructs expressed at similar levels, appeared identical on an SDS-PAGE gel, and exhibited monodisperse behavior on a size-exclusion chromatography column suggesting that the mutation did not disrupt the structure of the β-prism domain.

To further confirm that mutation of the key sugar binding aspartic acid did not disrupt the folding or stability of the protein, we subjected RbmC2 WT and RbmC2 D853A β-prism domains to a thermal melt monitored via circular dichroism (CD). CD spectra for RbmC2 WT and RbmC2 D853A before melting (at 20°C) exhibited similar profiles (S7A Fig) and estimated secondary structure composition (calculated with β-structure selection (BeStSel) [40], S7B Fig), also consistent with the RbmC2 crystal structure. RbmC2 WT and RbmC2 D853A maintained mostly β-strand secondary structure through 96°C and fitting with BeStSel showed an increase in parallel and left-twisted antiparallel β-sheets, suggesting that unfolding likely led to the formation of amyloid structures (S7B Fig). Although RbmC2 D853A displayed decreased CD signal beyond 54°C (perhaps due to lower solubility caused by the D853A mutation or a different high-temperature state), our results suggest that mutation of the aspartic acid residue did not destabilize the RbmC2 domain at temperatures below 50°C (S7C Fig), confirming validity of the blood binding assays and ITC, which were performed at room temperature. Due to the low solubility of the RbmC1 domain following cleavage from the GFP_UV_ fusion protein, we did not perform a CD melt on the RbmC1 mutant. Both RbmC1 WT and RbmC1 D539A fusion proteins expressed and purified in a soluble form and led to monodisperse peaks on a sizing column suggesting that like RbmC2, they adopt a folded structure at room temperature.

**Fig 6:**
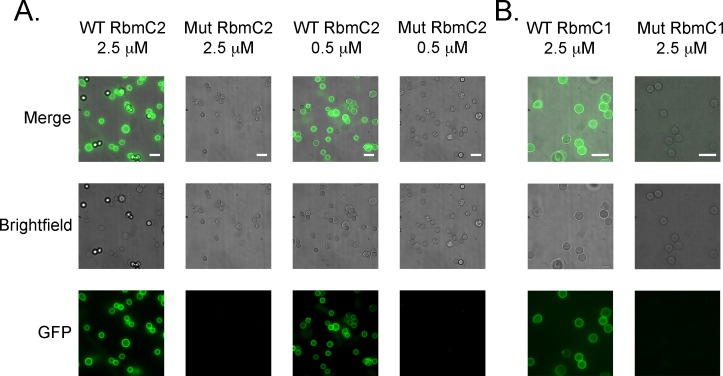
Binding of RbmC1 and RbmC2 to mammalian cells. (A) Fluorescence microscopy of defibrinated rabbit whole blood incubated with the RbmC2-GFP_UV_ fusion. Wild-type or a D853A point mutation (with significantly reduced glycan-binding activity) are shown. Images include GFP, brightfield, and merged channels for constructs at 2.5 μM and 0.5 μM concentrations. Each WT/mutant pair was placed on an identical brightness scale, but WT and D853A are on different brightness scales. The white scale bar (lower right on the merged image) represents 10 microns. (B) Same as in A, but with WT RbmC1 and the D539A point mutation (purple asterisk on Fig 1C).

Fluorescence images showed binding of both the RbmC1 and RbmC2 wild-type fusions to the rabbit blood cells (Fig 6A and 6B), while binding of the aspartic acid substitution mutants was not detected. RbmC1 and RbmC2 clearly recognized and bound tightly to mammalian blood cells known to display complex *N*-glycans in a specific manner (dependent on interactions with D539/D853), suggesting that RbmC1 and RbmC2 domains have the ability to target mammalian cell-surface glycans with high-affinity.

## Discussion

Our results indicate that the β-prism domains of VCC and RbmC share a strong preference for complex *N*-glycans and are likely directed against similar targets, whether in the environment or in the host. Similar to results seen with VCC [11], screening against the glycan library failed to show binding of RbmC1 and RbmC2 to high-mannose type glycans, suggesting that vibrio β-prism domains prefer glycans highly represented on mammalian cell surfaces [29,41]. Our protein-glycan complex structures illustrate the basis for this selectivity. The vibrio β-prism domain sugar-binding pocket is able to accommodate branching of carbohydrate moieties extending from the α1,3-linked mannose and a select subset of carbohydrate extensions from the α1,6-linked mannose of the mannotriose core. In complex *N*-glycans, the GlcNAc moiety attaches to the O2-position of the core α1,6-linked mannose [30], a configuration tolerated by the vibrio β-prism binding pocket. In branched high-mannose glycans, α1,3- and α1,6- linkages to the α1,6-linked mannose of the mannotriose core are utilized, both of which are sterically incompatible with the vibrio β-prism domain glycan binding pocket. Complex *N*-glycans with more than two antennae on the α1,6-linked mannose residue are also unlikely to bind, because these additional branches extend with β1,4- and β1,6-linkages, and both positions are also sterically blocked in the sugar binding pocket. Furthermore, *Vibrio cholerae* VPS consists of modified gulose, glucose and galactose monomers linked in α- and β1,4-linkages [33]. This linear polymer conformation is distinct from the bent shape made by α1,6 and α1,3-linkages in the mannotriose core and lacks the O4 position necessary for crucial hydrogen-bonding interactions with the key aspartic acid position in the β-prism domain. Therefore, we expect that VPS is sterically unlikely to bind to VCC and RbmC β-prism domains.

We were unable to express the β-prism domain from the biofilm matrix protein Bap1 and therefore cannot comment on whether Bap1 has a similar preference for complex *N*-glycans as VCC and RbmC. While Bap1 maintains the key aspartate residue (D348) and ring-stacking tryptophan (W387) that anchor the mannotriose ligand in RbmC2 (S2 Fig), the core tryptophan and leucine residues (W706/L707 in VCC, W948/L949 in RbmC2) are missing in Bap1 and instead replaced by a seven-residue insertion. RbmC and Bap1 have similar, but not identical roles in biofilm formation [21,22] and could thus target a different assortment of glycan receptors. This may involve the attachment of growing colonies to a host cell surface, or the absorption of clusters of cells already surrounded by biofilms to host surfaces in the gut. To the best of our knowledge, the majority of experiments involving biofilm formation using *V*. *cholerae* strains are performed on glass coverslips, glass liquid culture tubes, polystyrene plates or Teflon surfaces, all of which are unlikely to contain *N*-glycan anchor points. Under these conditions, we would not expect β-prism domains to be utilized in making adhesive contacts. Future experiments investigating biofilms grown on cultured cells or tissue surfaces may uncover variations in how the biofilm matrix forms, particularly when these additional adhesive interactions are part of the complex system.

Our structural and functional results suggest a model for *N*-glycan recognition by vibrio β-prism domains. Rather than targeting motifs on antennae termini that may vary heterogeneously across different cell types, they selectively bind to the invariant core. This arrangement is also less likely to be affected by further modifications, like core fucosylation of the *N*-acetylglucosamine stem [42]. Some glycans have an additional bisecting *N*-acetylglucosamine residue projecting out of the central mannose in the mannotriose core with a β1,4-linkage [42]. The orientation in which this core is bound in RbmC2 leaves little space for this modification, although binding could occur if W948 flips to a different rotamer. Plant complex *N*-glycans often contain an additional β1,2-xylose modification on the central mannose of the mannotriose core [28]. This position is relatively unhindered in our structures (Fig 4B), raising the possibility that vibrio biofilms could attach to plant surfaces, although these glycans were not represented on the mammalian glycan chip. By accommodating these and other modifications, the β-prism lectin may help toxins and biofilm proteins target a wider range of glycan structures in the environment and in hosts. Because the core structure described in this study is shared in all complex *N*-glycans, it would also be nearly impossible for a host to evolve resistance without substantial truncation of cell-surface glycans.

Our results also indicate that the affinity for *N*-glycans varies among the different vibrio β-prism domains. We determined the affinity for mannotriose and heptasaccharide glycan cores to be at least 100-fold stronger for RbmC β-prism domains than for the VCC β-prism domain. This 100-fold difference theoretically requires approximately 2.7 kcal/mol of binding energy, which can be explained by the presence of one or two additional hydrogen bonds or <150 Å^2^ of newly buried surface energy. The increased binding energy required for the 100-fold difference observed between RbmC and VCC β-prism domains can be accounted for by the additional hydrogen bonding interaction of Y894 (Y575 in RbmC1, F652 in VCC), the additional surface area provided by W896 (W577 in RbmC1, Y654 in VCC), or the additional hydrogen bond facilitated by T870 in RbmC2. VCC lyses cells at picomolar concentrations, so comparing these affinities, we expect RbmC to bind very tightly to cell surfaces containing *N*-glycan groups, particularly due to avidity effects from having two β-prism domains. Bap1, with only one β-prism domain, may bind less strongly. A mutant form of RbmC missing the C-terminal β-prism domain (RbmC2) is still able to rescue biofilm formation by a Δ*rbmC*/Δ*bap1* double mutant [21], likely due to the redundant nature of the β-prism domain. Both Bap1, with a single β-prism domain, and full-length RbmC are also able to rescue biofilm formation in a double knockout [12].

Our results demonstrate a common mechanism by which vibrio biofilm and toxin proteins utilize complex *N*-glycans to attach to and attack host cell membranes. While the role of biofilm attachment to complex *N*-glycans in the disease cycle has not yet been explored, it is possible that clusters of biofilm-encapsulated bacteria could strongly attach to the human intestinal epithelium following ingestion helping to seed new colonies. Or, newly established colonies in the gut could utilize epithelial surfaces as biofilm-dependent anchors. Interestingly, biofilm formation is negatively regulated by quorum-sensing pathways [43,44], suggesting that it may be adventitious to form biofilms during early stages of colonization, but not so when the pathogen prepares to leave the host. At the same time, VCC could help defend against immune cells [10,45] or cause localized inflammation by attacking the epithelial surface directly before quorum sensing eventually leads to the down-regulation of VCC [46] and activation of classical cholera toxin and other important virulence factors [47].

A better understanding of the role fulfilled by these glycan-interactions is important for devising interventions to block intoxication and biofilm formation by bacterial pathogens and may provide additional methods to target specific cell types. For example, cancer cells often display modified glycan structures on their cell membranes [48], a marker that might be exploited by agents specifically targeted against these glycans. These results also suggest further experiments aimed at studying biofilm formation on cellular substrates, where carbohydrate adhesion interactions may further modify the growing biofilm matrix.

## Materials and methods

### Glycan materials

Glycans used in this paper: methyl α-D-mannopyranoside (Sigma, M6882), *N*-acetyl-D-lactosamine (Carbosynth, OA08244), *N*-acetylglucosaminyl-β-1,2-mannose (Dextra, M292), 1,3-α-1,6-α-D-mannotriose (Carbosynth, OM05762 or Dextra, M336), *N*-linked core pentasaccharide (Dextra, M592), NGA2 *N*-linked heptasaccharide (Prozyme, GKC-004300), NA2 *N*-linked polysaccharide (Prozyme, GKC-024300), A2 *N*-linked polysaccharide (Prozyme, GKC-224300), asialofetuin (Sigma, A4781).

### Strains used in this study

NEB5α *E*. *coli* (C2987I), Shuffle T7 *E*. *coli* (C3026J), and T7 Express *E*. *coli* (C2566I). All strains were obtained from New England Biolabs, Ipswich, MA.

### Construction of RbmC and VCC β-prism lectin constructs

Full-length RbmC and Bap1 were cloned by PCR from *Vibrio cholerae* El Tor strain N16961 genomic DNA into the pET28b vector (Novagen, Inc.). Individual β-prism lectin domains (denoted RbmC1 and RbmC2, from residues S505 to T640 and S823 to Y957, respectively) were cloned into the pNGFP-BC vector to form GFP_UV_ fusion proteins [49], confirmed by DNA sequencing, and transformed into T7 Express *E*. *coli* for expression. The full-length VCC gene (*hlyA*, from *Vibrio cholerae* O1 El Tor strain 8731) [50], the VCC β-prism lectin domain cloned into the pET-28b vector [11], and the RbmC2 β-prism domain in pNGFP-BC were subjected to site-directed mutagenesis using a previously described procedure [51]. Briefly, complementary primers containing the desired mutation were used to amplify the entire gene-containing plasmid by PCR and the resulting DNA was digested by DpnI to remove parental DNA. The reaction was then transformed into NEB5α-cells. The resulting clones were miniprepped and sequenced to confirm the introduction of the site-directed mutation.

### Expression and purification of β-prism lectin and VCC full-length constructs

For expression of the RbmC1 and RbmC2 β-prism domains, LB broth supplemented with 100 mg/L ampicillin was inoculated with overnight cultures (1:60 dilution, v/v), grown at 37°C to an O.D. of 0.6, induced with 1 mM IPTG, and incubated at 30°C for 4 hours. Cells were pelleted by centrifugation at 3500 x g in a Sorvall LYNX 6000 centrifuge (F9-6x1000 LEX rotor) and lysed by passing three times through an Emulsiflex-C5 high-pressure homogenizer (Avestin, Inc.). The lysate was cleared at 40,000 x g for 30 minutes at 4°C (F20-12x50 LEX rotor) and the resulting supernatant loaded onto a 10 ml TOYOPEARL AF-Chelate-650M nickel column (Tosoh Corporation) equilibrated in TBS buffer (20 mM Tris pH 7.6, 150 mM NaCl). The column was washed with 10 column volumes of TBS buffer containing 40 mM imidazole and the protein eluted in TBS buffer containing 250 mM imidazole. To remove the GFP tag, the fusion proteins were incubated with 1:100 (wt/wt) human α-thrombin (Haematologic Technologies) for 4 hours at room temperature or 1:500 (wt/wt) trypsin (Sigma Aldrich) for 1 hour at room temperature and the reaction stopped with 20 mM EDTA and 1 mM AEBSF. Wild-type RbmC β-prism domains were separated from the polyhistidine-tagged GFP fusion partner by passing the over a Sepharose S6 10/300 size-exclusion column (GE Healthcare) in TBS buffer. RbmC2 D853A was separated from cleaved GFP by passage over a Superdex 200 Increase 10/300 column (GE Healthcare) in TBS buffer. VCC full-length and VCC β-prism lectin domain constructs were expressed and purified similarly, with the following modifications. VCC full-length protein was expressed in Shuffle T7 *E*. *coli* cells (New England Biolabs) for 4 hours at 30°C and the VCC β-prism lectin domain was expressed for 8 hours at 37°C in T7 Express *E*. *coli* cells. Both VCC proteins were purified over a 5-ml HisTrap Ni-NTA column (GE Healthcare) followed by a Superdex 200 10/300 size-exclusion column (GE Healthcare) run in TBS buffer.

### Glycan chip assay

Purified RbmC1 and RbmC2 GFP_UV_ fusions were fluorescently labeled by primary amine chemistry using an AlexaFluor 488 succinimidyl ester reagent (Thermo Fisher Scientific). Proteins were concentrated to 5.5 or 3.4 mg/ml and diluted into 100 mM sodium bicarbonate buffer, pH 9.0. While stirring, 0.15 mg of the dye (resuspended in 0.15 ml DMSO) was added to 1.1 ml of protein and incubated for 1 hour at room temperature. Unreacted dye was bound by adding 0.1 ml 1.5 M Tris pH 8.5 and removed from labeled proteins by running over a Superose 6 10/300 size exclusion column equilibrated in TBS buffer with 1mM EDTA and 1mM sodium azide. Labeled proteins were sent to Core H of the CFG for analysis against the mammalian glycan screen v. 5.2. In brief, glycan chips were incubated with 180 μg/ml of labeled lectin for 1 hour, washed three times to remove non-specific binding, and dried under nitrogen before imaging using a Perkin Elmer ProScanArray XL4000 scanner. The data are reported as the average response units of six replicates after removing the highest and the lowest data points. The entire dataset is freely available through the CFG website (www.functionalglycomics.org).

### Fluorescence binding assay

VCC β-prism lectin domain was buffer exchanged into PBS (20 mM sodium phosphate pH 7.4, 150 mM NaCl) by running over a Superdex 200 10/300 size exclusion column (GE Healthcare). Fluorescence assays were performed using a Fluoromax-2 spectrophotometer (Horiba Jobin-Yvon Inc.). Data were collected in 6 mm x 6 mm quartz cuvettes (Starna Cells, Inc.) continuously stirred throughout the span of the experiment. The intrinsic protein fluorescence was monitored by exciting protein samples at 295 nm with a band-pass of 4 x 4 nm. Upon addition of glycans (dissolved in PBS buffer), the increase in intrinsic fluorescence was measured and the fractional increase calculated and normalized to the highest point. All data were acquired in triplicate and the results plotted in Origin v. 8.0 (OriginLab Corporation) and fit using the RandoA function.

### Hemolysis assay

Purified VCC was activated by proteolytic cleavage using α-chymotrypsin (1:350 wt/wt) for 30 minutes at room temperature and serially diluted. Activated VCC dilutions were added to wells in a 96-well clear bottom plate containing defibrinated rabbit whole blood diluted to an absorbance at 595 nm of 1.0 in blood dilution buffer (20 mM sodium phosphate pH 7.4, 150 mM NaCl, 1mg/ml BSA). The absorbance was monitored at 595 nm every 15 seconds at room temperature in an iMark 96-well plate reader (Bio-rad Laboratories, Inc.). Raw absorbance data were converted into % lysis and HD_50_ values calculated as described previously [36] using KaleidaGraph v. 4.1.3 (Synergy Software).

### Isothermal calorimetry

Purified RbmC1-GFP_UV_ fusion, RbmC2-GFP_UV_ fusion, and VCC β-prism domains were dialyzed against TBS buffer overnight using a 3-kDa cutoff membrane. ITC data were collected using a MicroCal VP-ITC calorimeter at 25°C. Carbohydrates were dissolved in TBS and titrated (5 mM for mannotriose, *N*-acetyl-D-lactosamine, and *N*-acetylglucosaminyl-β-1,2-mannose; 120 μM for asialofetuin) into 1.44 ml of dialyzed protein (100 μM for VCC β-prism lectin domain, 48 μM for RbmC β-prism lectin domains). Blank subtracted ITC raw data were processed using NITPIC v 1.2.0 [52], analyzed with SEDPHAT v. 12.1b [53] and processed with GUSSI v. 1.0.8 (downloaded from http://biophysics.swmed.edu/MBR/software.html).

### Crystallization, X-ray data collection, and structure refinement

Purified RbmC2 lectin domain in TBS was concentrated to ~5 mg/ml using a 3 kDa cutoff Vivaspin concentrator (GE Healthcare Life Sciences). Crystals were grown using vapor diffusion in 24-well tissue culture plates by mixing 1:1 (v/v) protein and precipitant solution (0.1 M sodium acetate pH 4.6, 2M ammonium sulfate for all crystals) and suspending drops on siliconized cover slips over a 0.5 ml reservoir solution. For crystallization with ligands, a 1:10 molar excess of sugar was added to the RbmC2 protein solution. Apo and mannotriose X-ray diffraction data were collected on crystals (cryoprotected in mother liquor supplemented with 20% glycerol and flash-cooled in liquid nitrogen) using an Oxford Xcalibur Nova X-ray generator with an Onyx CCD detector (Oxford Diffraction) and indexed using CrysAliasPro (Rigaku Corporation) and Aimless [54]. GlcNAc-Man X-ray data were collected on a Rigaku HighFlux HomeLab system with a Raxis IV++ detector and processed using *iMOSFLM* [55]. Molecular replacement using the VCC β-prism lectin domain (PDB ID 1XEZ) as a search model was carried out by Phaser [56]. Multiple rounds of XYZ coordinate and individual *B*-factor refinement with phenix.refine [57] were interspersed with model building/rebuilding via Coot [58] using |2F_o_|—|F_c_| and |F_o_|—|F_c_| electron density maps. The progress of refinement was monitored by following the *R*_*work*_/*R*_*free*_ ratio (*R*_*free*_ consisting of 5–10% of reflections). Waters were selected and refined using the automated water picking feature of phenix.refine and ligands built into the electron density maps following the first round of rebuilding and refinement. Simulated annealing OMIT maps were constructed by removing the ligand from the final refined structure file, performing three macro-cycles of refinement with simulated annealing, and calculating a |F_o_|—|F_c_| map with phenix.maps. Occupancy refinement was carried out using phenix.refine for the GlcNAc-Man structure, which appeared to contain a mixture of the sugar and glycerol in the binding pocket. The two ligands were defined as alternate conformers and refined to a combined occupancy of 1.0 (final occupancies = 0.65 GlcNAc-Man, 0.35 glycerol).

To construct the RbmC2/pentasaccharide model, superimposed mannotriose and GlcNAc(β1–2)Man structures (which share an overlapping mannose) were used as a template to build the Man_3_GlcNAc core and a second GlcNAc residue built in from scratch. Five macro-cycles of geometry minimization and regularization were carried out using the phenix.geometry_minimization feature of Phenix [59]. Buried surface area calculations were performed using PISA [37] as implemented in Coot [58]. Contacts were determined using LigPlot+ v. 1.4.5 [60] with hydrogen bonds filtered using a cutoff of 3.35 Å. Model quality statistics were calculated using the MolProbity server accessed at http://molprobity.biochem.duke.edu.

### Circular dichroism (CD)

Trypsin-cleaved RbmC2 WT and RbmC2 D853A domains were separated from the GFP_UV_ fusion protein on Superose 6 and Sephadex 200 10/300 Increase size exclusion columns, respectively, in TBS. Samples were concentrated using a Vivaspin 5 kDa-cutoff centrifugal concentrator to a concentration of 340 μM and diluted to a final concentration of 12.5 μM with 10 mM sodium phosphate buffer pH 7.4. Samples were loaded into 0.2 cm quartz cuvettes (Cole-Parmer, Staffordshire, UK). CD data were collected on a Jasco J-810 spectrometer (Jasco Inc., Easton, MD). Thermal denaturation was monitored using the change in molar extinction at 222 nm while changing the temperature in 2 degree increments from 20°C to 96°C. Tm values were calculated from the mid-point of sigmoidal fits to the temperature data. Secondary structure composition was estimated using the β-structure selection server (BeStSel, http://bestsel.elte.hu) using CD data from 200–250 nm [40].

### Fluorescence microscopy

Defibrinated whole rabbit blood (Remel, ThermoFisher Scientific) was washed once with PBS buffer and resuspended cells incubated with a 0.5 and 2.5 μM concentrations of purified RbmC2-GFP_UV_ for 5 minutes on ice, or a 2.5 μM concentration of RbmC1-GFP_UV_. As a control for non-specific binding, a point mutation shown to significantly reduce glycan binding was used as a control (D853A in RbmC2 and D539A in RbmC1). Cells were centrifuged at 500 x g in a microcentrifuge tube for 5 minutes to pellet cells, the supernatant removed, and the cells gently resuspended in 25 μL of PBS buffer. Bright-field and fluorescence microscopy images were acquired using a DeltaVision RT imaging system (Applied Precision) adapted to an Olympus (IX71) microscope. Images were acquired with a fixed exposure time (3 s for brightfield and 4 s for fluorescence) so that samples could be compared on the same intensity scales. Z-stack sections of 0.5 μm were collected using a 60X or 100X objective and images put on identical scales and superimposed using the Fiji distribution of Image J [61]. For GFP fluorescence images, excitation and emission filter wavelengths were 490 and 528 nm, respectively.

## Supporting information

S1 Fig Representative fluorescence and ITC binding data. (A) Tryptophan-fluorescence binding curve for NGA2 binding to isolated RbmC2. Data were fit to a RandoA function in Origin v. 8.0 and error bars represent the standard error of the mean from three replicates. ITC binding data for WT RbmC2 binding to (B) mannotriose and (C) asialofetuin. Asialofetuin is heterogeneously and multiply glycosylated, and was therefore fit using 3 sites per glycoprotein; the protein concentration was input for fitting.
(TIF)

S2 Fig Comparison of VCC, RbmC1, RbmC2, and Bap1 equivalent positions. Positions conserved in all four *V*. *cholerae* β-prism domains (VCC, RbmC1, RbmC2, Bap1) are in red type based on multiple sequence alignments. Positions with asterisks were mutated in this study. The type of contact made with mannotriose or GlcNAc-Man structures is noted with dashes representing residues not contacting ligands. Contacts were determined using LigPlot+ v. 1.4.5 with hydrogen bonds filtered using a cutoff of 3.35 Å and hydrophobic interactions filtered at 3.9 Å. Also listed is whether the interaction primarily involves a peptide backbone hydrogen-bonding interaction, side-chain hydrogen-bonding interaction, or hydrophobic van der Waals (vdW) interaction. The fold loss in VCC hemolytic activity when mutated to the amino acid shown in parenthesis is also noted (see Fig 5A).
(TIF)

S3 Fig RbmC2/mannotriose contacts. Schematic representation of hydrogen-bonding and hydrophobic contacts between RbmC2 and the mannotriose ligand. Hydrogen bonds are shown as blue dotted lines and hydrophobic interactions by red arcs. A cartoon schematic of a typical complex biantennary *N*-glycan with mannotriose boxed is shown (lower left).
(TIF)

S4 Fig RbmC2/GlcNAc-Man contacts. Schematic representation of hydrogen-bonding and hydrophobic contacts between RbmC2 and the GlcNAc-Man ligand.
(TIF)

S5 Fig Comparison between RbmC2 apo, GlcNAc-Man, and mannotriose-bound structures. (A) Superposition between mannotriose, GlcNAc-Man, and apo structures (chain B, one of three chains in the asymmetric unit). The PVQGT loop of only one asymmetric unit domain is structured, adopting an alternative conformation to the mannotriose-bound structure loop. The composite GlcNAc-mannotriose glycan fragment is shown in a green and blue stick representation. (B) Superposition between apo (one copy), GlcNAc-Man, and mannotriose-bound RbmC2 structures illustrating slight aromatic side-chain movements that occur upon ligand binding.
(TIF)

S6 Fig ITC for RbmC2 WT and D853A mutant. (A) ITC isotherm of asialofetuin binding to purified GFP_UV_-RbmC2 fusion produced by GUSSI. (B) Binding of the D853A point mutant GFP_UV_-RbmC2 fusion under identical conditions illustrating loss of asialofetuin affinity.
(TIF)

S7 Fig CD melt of RbmC2 WT and D853A mutant. (A) Superposition of CD spectra for WT (left) and D853A (right) RbmC2 including scans for every 6°C in folded and melted ranges, every 2°C in transition range to illustrate variable unfolding pathway between 50–60°C. (B) Table illustrating percentages of secondary structure elements for each protein at 20°C and 96°C as calculated by BeStSel. Calculation of secondary structure from the RbmC2 apo crystal structure using DSSP [65] indicates 2.2% α-helix, 52.9% β-sheet, and 44.9% other. The antiparallel β-sheet category is broken down into left-twisted, relaxed, and right-twisted sheets. (C) Plot showing unfolding of RbmC2 WT and RbmC2 D853A proteins based on the CD melt data from 34 to 76°C. The mutant data is presented both as % unfolded based on the absolute shift of the CD signal and on the cumulative CD signal change from folded to unfolded states (a dip and then rise in the CD signal at 222 nm). From a sigmoidal fit of the curves, the T_m_ is estimated to be 55°C for WT and 54°C for the mutant.
(TIF)

## References

[1] Rostand KS, Esko JD. Microbial adherence to and invasion through proteoglycans. Infect Immun. 1997;65: 1–8. 8975885 10.1128/iai.65.1.1-8.1997 PMC174549

[2] Esko JD, Sharon N. Microbial Lectins: Hemagglutinins, Adhesins, and Toxins In: Varki A, Cummings RD, Esko JD, Freeze HH, Stanley P, Bertozzi CR, et al, editors. Essentials of Glycobiology. 2nd ed. Cold Spring Harbor (NY): Cold Spring Harbor Laboratory Press; 2009 pp 489–500. 20301238

[3] Kato K, Ishiwa A. The role of carbohydrates in infection strategies of enteric pathogens. Trop Med Health. 2015;43: 41–52. doi: 10.2149/tmh.2014-25 25859152 10.2149/tmh.2014-25 PMC4361345

[4] Drickamer K, Taylor ME. Glycan arrays for functional glycomics. Genome Biol. 2002;3: reviews 1034. 1-reviews1034.4 10.1186/gb-2002-3-12-reviews1034 PMC151192 12537579

[5] Sharon N. Carbohydrates as future anti-adhesion drugs for infectious diseases. Biochim Biophys Acta. 2006;1760(4): 527–537. doi: 10.1016/j.bbagen.2005.12.008 16564136 10.1016/j.bbagen.2005.12.008

[6] Balzarini J. Targeting the glycans of glycoproteins: a novel paradigm for antiviral therapy. Nat Rev Microbiol. 2007;5(8): 583–597. doi: 10.1038/nrmicro1707 17632570 10.1038/nrmicro1707 PMC7098186

[7] Sato S, Ouellet M, St-Pierre C, Tremblay MJ. Glycans, galectins, and HIV-1 infection. Ann N Y Acad Sci. 2012;1253: 133–148. doi: 10.1111/j.1749-6632.2012.06475.x 22524424 10.1111/j.1749-6632.2012.06475.x

[8] Merritt EA, Sarfaty S, van den Akker F, L'Hoir C, Martial JA, Hol WG. Crystal structure of cholera toxin B-pentamer bound to receptor GM1 pentasaccharide. Protein Sci. 1994;3(2): 166–175. doi: 10.1002/pro.5560030202 8003954 10.1002/pro.5560030202 PMC2142786

[9] Olivier V, Haines GK 3rd, Tan Y, Satchell KJ. Hemolysin and the multifunctional autoprocessing RTX toxin are virulence factors during intestinal infection of mice with *Vibrio cholerae* El Tor O1 strains. Infect Immun. 2007;75(10): 5035–5042. doi: 10.1128/IAI.00506-07 17698573 10.1128/IAI.00506-07 PMC2044521

[10] Queen J, Satchell KJ. Neutrophils Are Essential for Containment of *Vibrio cholerae* to the Intestine during the Proinflammatory Phase of Infection. Infect Immun. 2012;80(8): 2905–2913. doi: 10.1128/IAI.00356-12 22615254 10.1128/IAI.00356-12 PMC3434586

[11] Levan S, De S, Olson R. *Vibrio cholerae* cytolysin recognizes the heptasaccharide core of complex *N*-glycans with nanomolar affinity. J Mol Biol. 2013;425(5): 944–957. doi: 10.1016/j.jmb.2012.12.016 23274141 10.1016/j.jmb.2012.12.016 PMC3578121

[12] Fong JC, Yildiz FH. The rbmBCDEF gene cluster modulates development of rugose colony morphology and biofilm formation in *Vibrio cholerae*. J Bacteriol. 2007;189(6): 2319–2330. doi: 10.1128/JB.01569-06 17220218 10.1128/JB.01569-06 PMC1899372

[13] Moorthy S, Watnick PI. Identification of novel stage-specific genetic requirements through whole genome transcription profiling of *Vibrio cholerae* biofilm development. Mol Microbiol. 2005;57(6): 1623–1635. doi: 10.1111/j.1365-2958.2005.04797.x 16135229 10.1111/j.1365-2958.2005.04797.x PMC2600799

[14] Watnick PI, Kolter R. Steps in the development of a *Vibrio cholerae* El Tor biofilm. Mol Microbiol. 1999;34(3): 586–595. doi: 10.1046/j.1365-2958.1999.01624.x 10564499 10.1046/j.1365-2958.1999.01624.x PMC2860543

[15] Hobley L, Harkins C, MacPhee CE, Stanley-Wall NR. Giving structure to the biofilm matrix: an overview of individual strategies and emerging common themes. FEMS Microbiol Rev. 2015;39(5): 649–669. doi: 10.1093/femsre/fuv015 25907113 10.1093/femsre/fuv015 PMC4551309

[16] Teschler JK, Zamorano-Sánchez D, Utada AS, Warner CJ, Wong GC, Linington RG, et al Living in the matrix: assembly and control of *Vibrio cholerae* biofilms. Nat Rev Microbiol. 2015;13(5): 255–268. doi: 10.1038/nrmicro3433 25895940 10.1038/nrmicro3433 PMC4437738

[17] Yildiz FH, Schoolnik GK. *Vibrio cholerae* O1 El Tor: identification of a gene cluster required for the rugose colony type, exopolysaccharide production, chlorine resistance, and biofilm formation. Proc Natl Acad Sci U S A. 1999;96(7): 4028–4033. 10097157 10.1073/pnas.96.7.4028 PMC22414

[18] Huq A, Xu B, Chowdhury MA, Islam MS, Montilla R, Colwell RR. A simple filtration method to remove plankton-associated *Vibrio cholerae* in raw water supplies in developing countries. Appl Environ Microbiol. 1996;62(7): 2508–2512. 8779590 10.1128/aem.62.7.2508-2512.1996 PMC168033

[19] Faruque SM, Biswas K, Udden SM, Ahmad QS, Sack DA, Nair GB, et al Transmissibility of cholera: in vivo-formed biofilms and their relationship to infectivity and persistence in the environment. Proc Natl Acad Sci USA. 2006;103(16): 6350–6355. doi: 10.1073/pnas.0601277103 16601099 10.1073/pnas.0601277103 PMC1458881

[20] Reichhardt C, Fong JC, Yildiz F, Cegelski L. Characterization of the *Vibrio cholerae* extracellular matrix: a top-down solid-state NMR approach. Biochim Biophys Acta. 2015;1848(1 Pt B): 378–383. doi: 10.1016/j.bbamem.2014.05.030 24911407 10.1016/j.bbamem.2014.05.030 PMC4406247

[21] Absalon C, Van Dellen K, Watnick PI. A communal bacterial adhesin anchors biofilm and bystander cells to surfaces. PLoS Pathog. 2011;7(8): e1002210 doi: 10.1371/journal.ppat.1002210 21901100 10.1371/journal.ppat.1002210 PMC3161981

[22] Berk V, Fong JC, Dempsey GT, Develioglu ON, Zhuang X, Liphardt J, et al Molecular architecture and assembly principles of *Vibrio cholerae* biofilms. Science. 2012;337(6091): 236–239. doi: 10.1126/science.1222981 22798614 10.1126/science.1222981 PMC3513368

[23] Yan J, Sharo AG, Stone HA, Wingreen NS, Bassler BL. *Vibrio cholerae* biofilm growth program and architecture revealed by single-cell live imaging. Proc Natl Acad Sci U S A. 2016;113(36): E5337–E5343. doi: 10.1073/pnas.1611494113 27555592 10.1073/pnas.1611494113 PMC5018804

[24] Geissner A, Anish C, Seeberger PH. Glycan arrays as tools for infectious disease research. Curr Opin in Chem Biol. 2014;18: 38–45. doi: 10.1016/j.cbpa.2013.11.013 24534751 10.1016/j.cbpa.2013.11.013

[25] Flannery A, Gerlach JQ, Joshi L, Kilcoyne M. Assessing Bacterial Interactions Using Carbohydrate-Based Microarrays. Microarrays. 2015;4(4): 690–713. doi: 10.3390/microarrays4040690 27600247 10.3390/microarrays4040690 PMC4996414

[26] Altschul SF, Gish W, Miller W, Myers EW, Lipman DJ. Basic local alignment search tool. J Mol Biol. 1990;215(3): 403–410. doi: 10.1016/S0022-2836(05)80360-2 2231712 10.1016/S0022-2836(05)80360-2

[27] Watnick PI, Kolter R. Steps in the development of a *Vibrio cholerae* El Tor biofilm. Mol Microbiol. 1999;34(3): 586–595. 10564499 10.1046/j.1365-2958.1999.01624.x PMC2860543

[28] Strasser R. Plant protein glycosylation. Glycobiology. 2016;26(9): 926–939. doi: 10.1093/glycob/cww023 26911286 10.1093/glycob/cww023 PMC5045529

[29] Marth JD, Grewal PK. Mammalian glycosylation in immunity. Nat Rev Immunol. 2008;8(11): 874–887. doi: 10.1038/nri2417 18846099 10.1038/nri2417 PMC2768770

[30] Stanley P, Schachter H, Taniguchi N. *N*-Glycans In: Varki A, Cummings RD, Esko JD, Freeze HH, Stanley P, Bertozzi CR, et al, editors. Essentials of Glycobiology. 2nd ed. Cold Spring Harbor (NY): Cold Spring Harbor Laboratory Press; 2009 pp 101–114.

[31] Park D, Brune KA, Mitra A, Marusina AI, Maverakis E, Lebrilla CB. Characteristic Changes in Cell Surface Glycosylation Accompany Intestinal Epithelial Cell (IEC) Differentiation: High Mannose Structures Dominate the Cell Surface Glycome of Undifferentiated Enterocytes. Mol Cell Proteomics. 2015;14(11): 2910–2921. doi: 10.1074/mcp.M115.053983 26355101 10.1074/mcp.M115.053983 PMC4638035

[32] Parry S, Hanisch FG, Leir SH, Sutton-Smith M, Morris HR, Dell A, Harris A. *N*-Glycosylation of the MUC1 mucin in epithelial cells and secretions. Glycobiology. 2006;16(7): 623–634. doi: 10.1093/glycob/cwj110 16585136 10.1093/glycob/cwj110

[33] Yildiz F, Fong J, Sadovskaya I, Grard T, Vinogradov E. Structural characterization of the extracellular polysaccharide from *Vibrio cholerae* O1 El-Tor. PLoS ONE. 2014;9(1): e86751 doi: 10.1371/journal.pone.0086751 24520310 10.1371/journal.pone.0086751 PMC3901696

[34] Fong JC, Rogers A, Michael AK, Parsley NC, Cornell WC, Lin YC, et al Structural dynamics of RbmA governs plasticity of *Vibrio cholerae* biofilms. Elife. 2017;6: e26163 doi: 10.7554/eLife.26163 28762945 10.7554/eLife.26163 PMC5605196

[35] Green ED, Adelt G, Baenziger JU, Wilson S, Van Halbeek H. The asparagine-linked oligosaccharides on bovine fetuin. Structural analysis of *N*-glycanase-released oligosaccharides by 500-megahertz 1H NMR spectroscopy. J Biol Chem. 1988;263(34): 18253–18268. 2461366

[36] De S, Bubnys A, Alonzo F 3rd, Hyun J, Lary JW, Cole JL, et al The Relationship between Glycan Binding and Direct Membrane Interactions in *Vibrio cholerae* Cytolysin, a Channel-forming Toxin. J Biol Chem. 2015;290(47): 28402–28415. doi: 10.1074/jbc.M115.675967 26416894 10.1074/jbc.M115.675967 PMC4653697

[37] Krissinel E, Henrick K. Inference of macromolecular assemblies from crystalline state. J Mol Biol. 2007;372(3): 774–797. doi: 10.1016/j.jmb.2007.05.022 17681537 10.1016/j.jmb.2007.05.022

[38] Chen VB, Arendall WB 3rd, Headd JJ, Keedy DA, Immormino RM, Kapral GJ, et al MolProbity: all-atom structure validation for macromolecular crystallography. Acta Crystallogr D Biol Crystallogr. 2010;66(Pt 1): 12–21. doi: 10.1107/S0907444909042073 20057044 10.1107/S0907444909042073 PMC2803126

[39] Sutton-Smith M, Wong NK, Khoo KH, Wu SW, Yu SY, Patankar MS, et al Analysis of protein-linked glycosylation in a sperm-somatic cell adhesion system. Glycobiology. 2007;17(6): 553–567. doi: 10.1093/glycob/cwm025 17337520 10.1093/glycob/cwm025

[40] Micsonai A, Wien F, Kernya L, Lee YH, Goto Y, Réfrégiers M, et al Accurate secondary structure prediction and fold recognition for circular dichroism spectroscopy. Proc Natl Acad Sci U S A. 2015;112(24): E3095–3103. doi: 10.1073/pnas.1500851112 26038575 10.1073/pnas.1500851112 PMC4475991

[41] Moremen KW, Tiemeyer M, Nairn AV. Vertebrate protein glycosylation: diversity, synthesis and function. Nat Rev Mol Cell Biol. 2012;13(7): 448–462. doi: 10.1038/nrm3383 22722607 10.1038/nrm3383 PMC3934011

[42] Takahashi M, Kuroki Y, Ohtsubo K, Taniguchi N. Core fucose and bisecting GlcNAc, the direct modifiers of the *N*-glycan core: their functions and target proteins. Carbohydr Res. 2009;344(12): 1387–1390. doi: 10.1016/j.carres.2009.04.031 19508951 10.1016/j.carres.2009.04.031

[43] Hammer BK, Bassler BL. Quorum sensing controls biofilm formation in *Vibrio cholerae*. Mol Microbiol. 2003;50(1): 101–104. 14507367 10.1046/j.1365-2958.2003.03688.x

[44] Waters CM, Lu W, Rabinowitz JD, Bassler BL. Quorum sensing controls biofilm formation in *Vibrio cholerae* through modulation of cyclic di-GMP levels and repression of *vpsT*. J Bacteriol. 2008;190(7): 2527–2536. doi: 10.1128/JB.01756-07 18223081 10.1128/JB.01756-07 PMC2293178

[45] Jeong HG, Satchell KJF. Additive function of *Vibrio vulnificus* MARTX_Vv_ and VvhA cytolysins promotes rapid growth and epithelial tissue necrosis during intestinal infection. PLoS Pathog. 2012;8(3): e1002581 doi: 10.1371/journal.ppat.1002581 22457618 10.1371/journal.ppat.1002581 PMC3310748

[46] Tsou AM, Zhu J. Quorum sensing negatively regulates hemolysin transcriptionally and posttranslationally in *Vibrio cholerae*. Infect Immun. 2010;78(1): 461–467. doi: 10.1128/IAI.00590-09 19858311 10.1128/IAI.00590-09 PMC2798175

[47] Zhu J, Miller MB, Vance RE, Dziejman M, Bassler BL, Mekalanos JJ. Quorum-sensing regulators control virulence gene expression in *Vibrio cholerae*. 2002;99(5): 3129–3134. doi: 10.1073/pnas.052694299 11854465 10.1073/pnas.052694299 PMC122484

[48] Pinho SS, Reis CA. Glycosylation in cancer: mechanisms and clinical implications. Nat Rev Cancer. 2015;15(9): 540–555. doi: 10.1038/nrc3982 26289314 10.1038/nrc3982

[49] Kawate T, Gouaux E. Fluorescence-detection size-exclusion chromatography for precrystallization screening of integral membrane proteins. Structure. 2006;14(4): 673–681. doi: 10.1016/j.str.2006.01.013 16615909 10.1016/j.str.2006.01.013

[50] Olson R, Gouaux E. Crystal structure of the *Vibrio cholerae* cytolysin (VCC) pro-toxin and its assembly into a heptameric transmembrane pore. J Mol Biol. 2005;350(5): 997–1016. doi: 10.1016/j.jmb.2005.05.045 15978620 10.1016/j.jmb.2005.05.045

[51] Braman J, Papworth C, Greener A. Site-directed mutagenesis using double-stranded plasmid DNA templates. Methods Mol Biol. 1996;57: 31–44. doi: 10.1385/0-89603-332-5:31 8849992 10.1385/0-89603-332-5:31

[52] Scheuermann TH, Brautigam CA. High-precision, automated integration of multiple isothermal titration calorimetric thermograms: new features of NITPIC. Methods. 2015;76: 87–98. doi: 10.1016/j.ymeth.2014.11.024 25524420 10.1016/j.ymeth.2014.11.024 PMC4380771

[53] Zhao H, Piszczek G, Schuck P. SEDPHAT—a platform for global ITC analysis and global multi-method analysis of molecular interactions. Methods. 2015;76: 137–148. doi: 10.1016/j.ymeth.2014.11.012 25477226 10.1016/j.ymeth.2014.11.012 PMC4380758

[54] Winn MD, Ballard CC, Cowtan KD, Dodson EJ, Emsley P, Evans PR, et al Overview of the CCP4 suite and current developments. Acta Crystallogr D Biol Crystallogr. 2011;67(Pt 4): 235–242. doi: 10.1107/S0907444910045749 21460441 10.1107/S0907444910045749 PMC3069738

[55] Battye TG, Kontogiannis L, Johnson O, Powell HR, Leslie AG. iMOSFLM: a new graphical interface for diffraction-image processing with MOSFLM. Acta Crystallogr D Biol Crystallogr. 2011;67(Pt 4): 271–281. doi: 10.1107/S0907444910048675 21460445 10.1107/S0907444910048675 PMC3069742

[56] McCoy AJ, Grosse-Kunstleve RW, Adams PD, Winn MD, Storoni LC, Read RJ. *Phaser* crystallographic software. J Appl Crystallogr. 2007;40(Pt 4): 658–674. doi: 10.1107/S0021889807021206 19461840 10.1107/S0021889807021206 PMC2483472

[57] Afonine PV, Grosse-Kunstleve RW, Echols N, Headd JJ, Moriarty NW, Mustyakimov M, et al Towards automated crystallographic structure refinement with phenix.refine. Acta Crystallogr D Biol Crystallogr. 2012;68(Pt 4): 352–367. doi: 10.1107/S0907444912001308 22505256 10.1107/S0907444912001308 PMC3322595

[58] Emsley P, Cowtan K. Coot: model-building tools for molecular graphics. Acta Crystallogr D Biol Crystallogr. 2004;60(Pt 12 Pt 1): 2126–2132. doi: 10.1107/S0907444904019158 15572765 10.1107/S0907444904019158

[59] Adams PD, Afonine PV, Bunkóczi G, Chen VB, Davis IW, Echols N, et al *PHENIX*: a comprehensive Python-based system for macromolecular structure solution. Acta Crystallogr D Biol Crystallogr. 2010 66: 213–221. doi: 10.1107/S0907444909052925 20124702 10.1107/S0907444909052925 PMC2815670

[60] Laskowski RA, Swindells MB. LigPlot+: multiple ligand-protein interaction diagrams for drug discovery. J Chem Inf Model. 2011;51(10): 2778–2786. doi: 10.1021/ci200227u 21919503 10.1021/ci200227u

[61] Schindelin J, Arganda-Carreras I, Frise E, Kaynig V, Longair M, Pietzsch T, et al Fiji: an open-source platform for biological-image analysis. Nat Methods. 2012;9(7): 676–682. doi: 10.1038/nmeth.2019 22743772 10.1038/nmeth.2019 PMC3855844

[62] De S, Olson R. Crystal structure of the *Vibrio cholerae* cytolysin heptamer reveals common features among disparate pore-forming toxins. Proc Natl Acad Sci U S A. 2011;108(18): 7385–7390. doi: 10.1073/pnas.1017442108 21502531 10.1073/pnas.1017442108 PMC3088620

[63] Kumar S, Stecher G, Tamura K. MEGA7: Molecular Evolutionary Genetics Analysis Version 7.0 for Bigger Datasets. Mol Biol Evol. 2016;33(7): 1870–1874. doi: 10.1093/molbev/msw054 27004904 10.1093/molbev/msw054 PMC8210823

[64] Robert X, Gouet P. Deciphering key features in protein structures with the new ENDscript server. Nucleic Acids Research. Oxford University Press; 2014;42: W320–4. doi: 10.1093/nar/gku316 24753421 10.1093/nar/gku316 PMC4086106

[65] Kabsch W, Sander C. Dictionary of protein secondary structure: pattern recognition of hydrogen-bonded and geometrical features. Biopolymers. 1983;22(12): 2577–2637. doi: 10.1002/bip.360221211 6667333 10.1002/bip.360221211

