# Supplementary figures and images for: Structural basis of mammalian glycan targeting by Vibrio cholerae cytolysin and biofilm proteins
Source: PLoS Pathog. 2018 Feb 12;14(2):e1006841. doi: 10.1371/journal.ppat.1006841 (PMC5825169; doi:10.1371/journal.ppat.1006841)

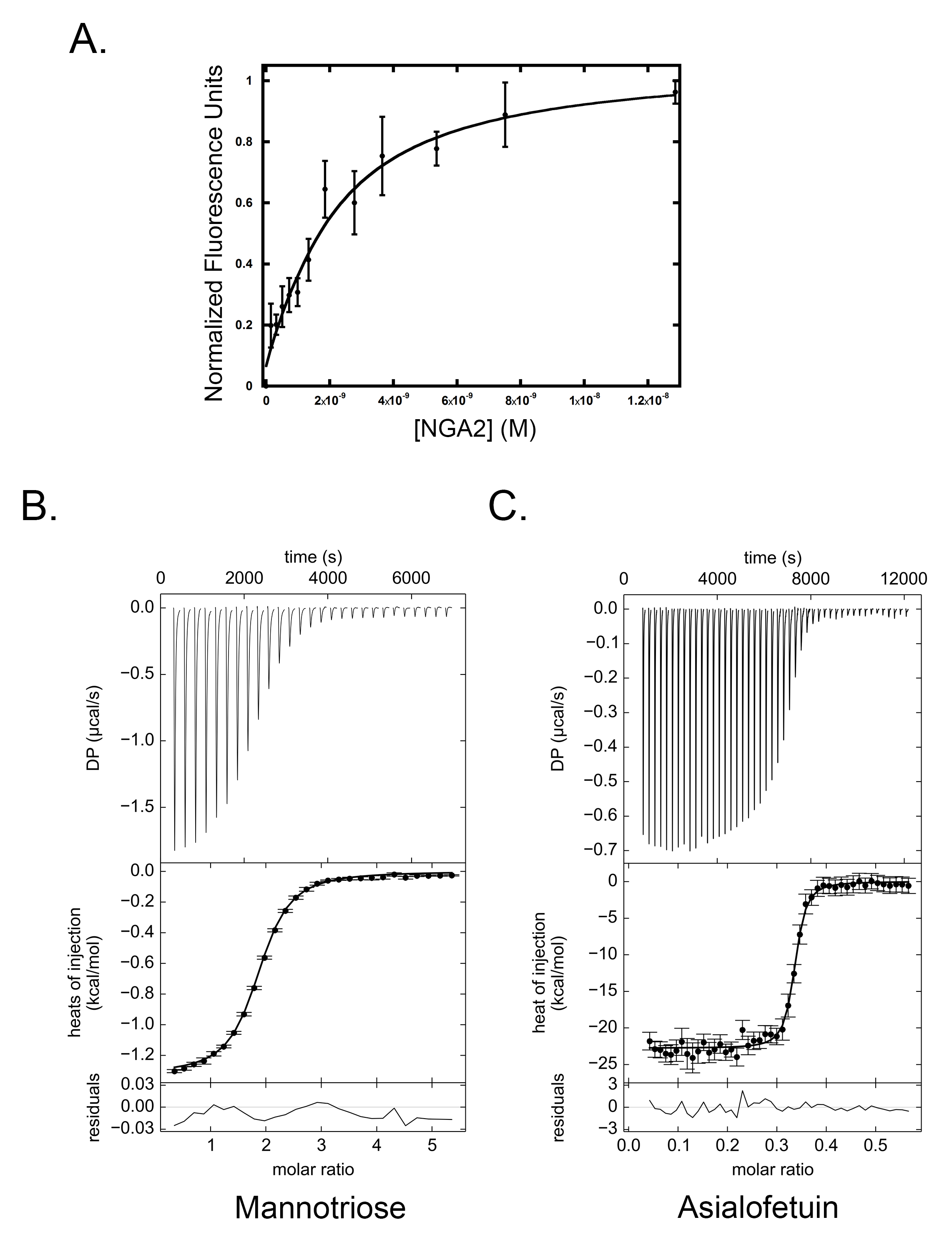

Supplement: S1 Fig — (A) Tryptophan-fluorescence binding curve for NGA2 binding to isolated RbmC2. Data were fit to a RandoA function in Origin v. 8.0 and error bars represent the standard error of the mean from three replicates. ITC binding data for WT RbmC2 binding to (B) mannotriose and (C) asialofetuin. Asialofetuin is heterogeneously and multiply glycosylated, and was therefore fit using 3 sites per glycoprotein; the protein concentration was input for fitting. (TIF) [file ppat.1006841.s001.tif]

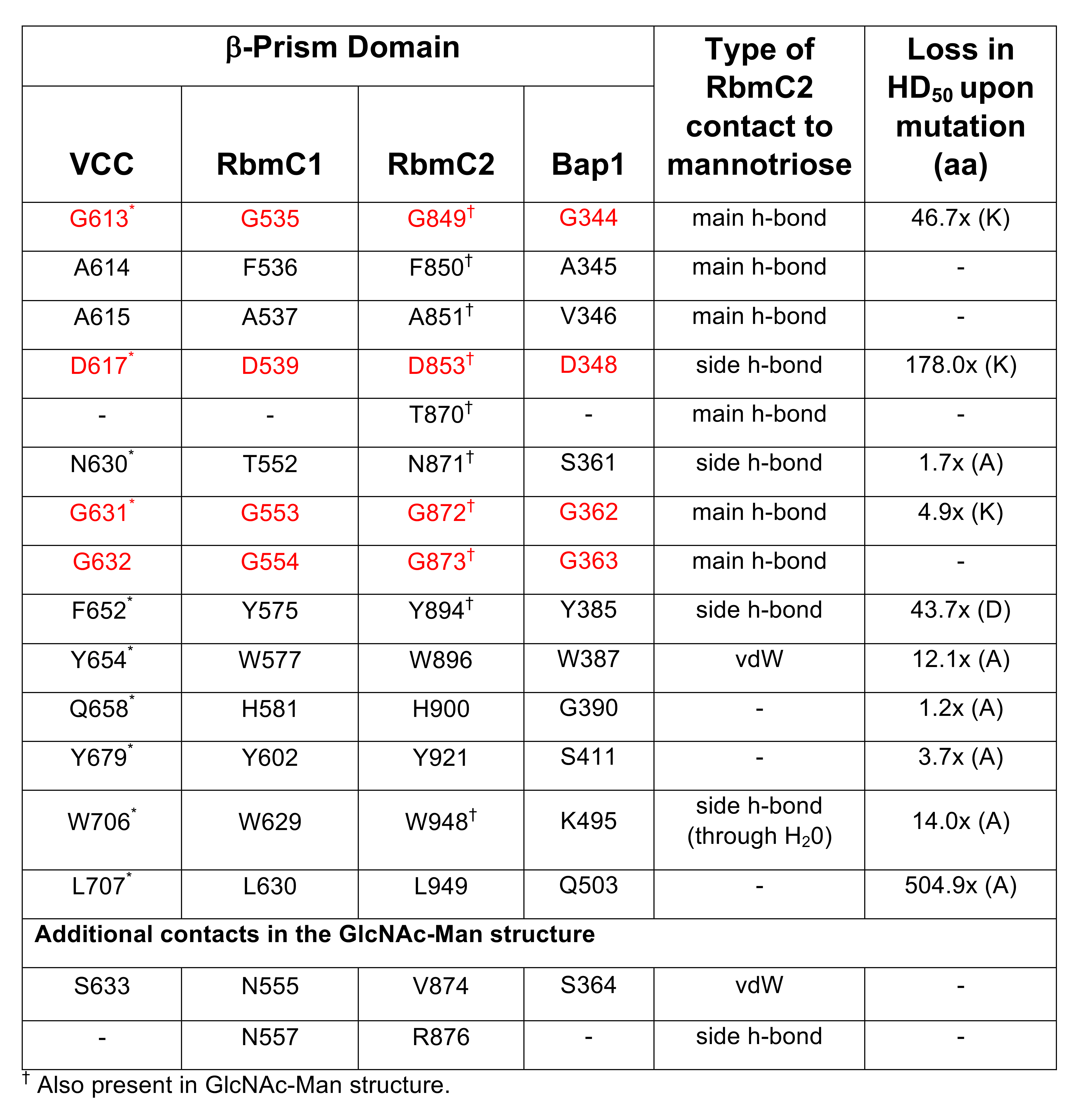

Supplement: S2 Fig — Positions conserved in all four V. cholerae β-prism domains (VCC, RbmC1, RbmC2, Bap1) are in red type based on multiple sequence alignments. Positions with asterisks were mutated in this study. The type of contact made with mannotriose or GlcNAc-Man structures is noted with dashes representing residues not contacting ligands. Contacts were determined using LigPlot+ v. 1.4.5 with hydrogen bonds filtered using a cutoff of 3.35 Å and hydrophobic interactions filtered at 3.9 Å. Also listed is whether the interaction primarily involves a peptide backbone hydrogen-bonding interaction, side-chain hydrogen-bonding interaction, or hydrophobic van der Waals (vdW) interaction. The fold loss in VCC hemolytic activity when mutated to the amino acid shown in parenthesis is also noted (see Fig 5A). (TIF) [file ppat.1006841.s002.tif]

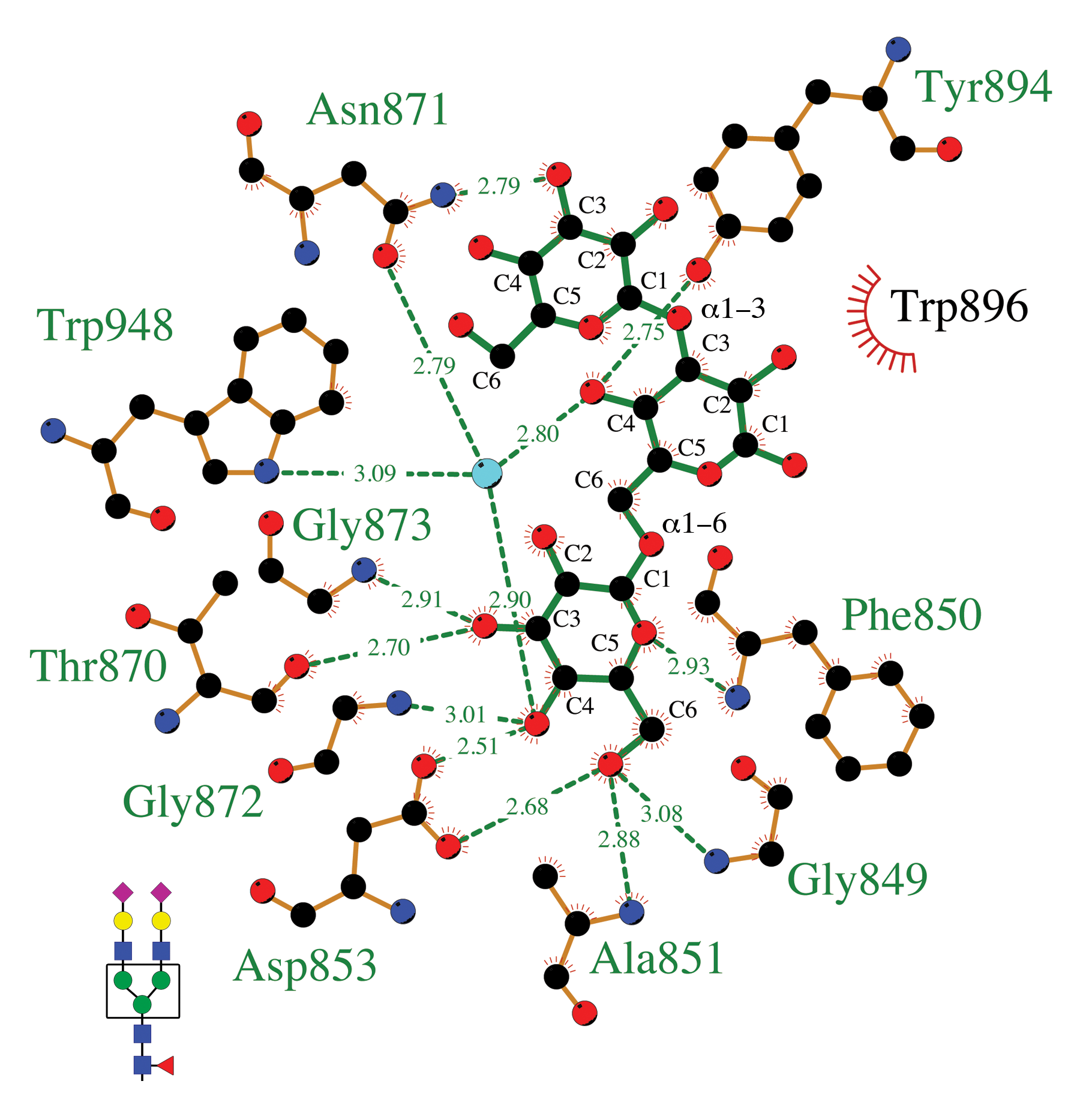

Supplement: S3 Fig — Schematic representation of hydrogen-bonding and hydrophobic contacts between RbmC2 and the mannotriose ligand. Hydrogen bonds are shown as blue dotted lines and hydrophobic interactions by red arcs. A cartoon schematic of a typical complex biantennary N-glycan with mannotriose boxed is shown (lower left). (TIF) [file ppat.1006841.s003.tif]

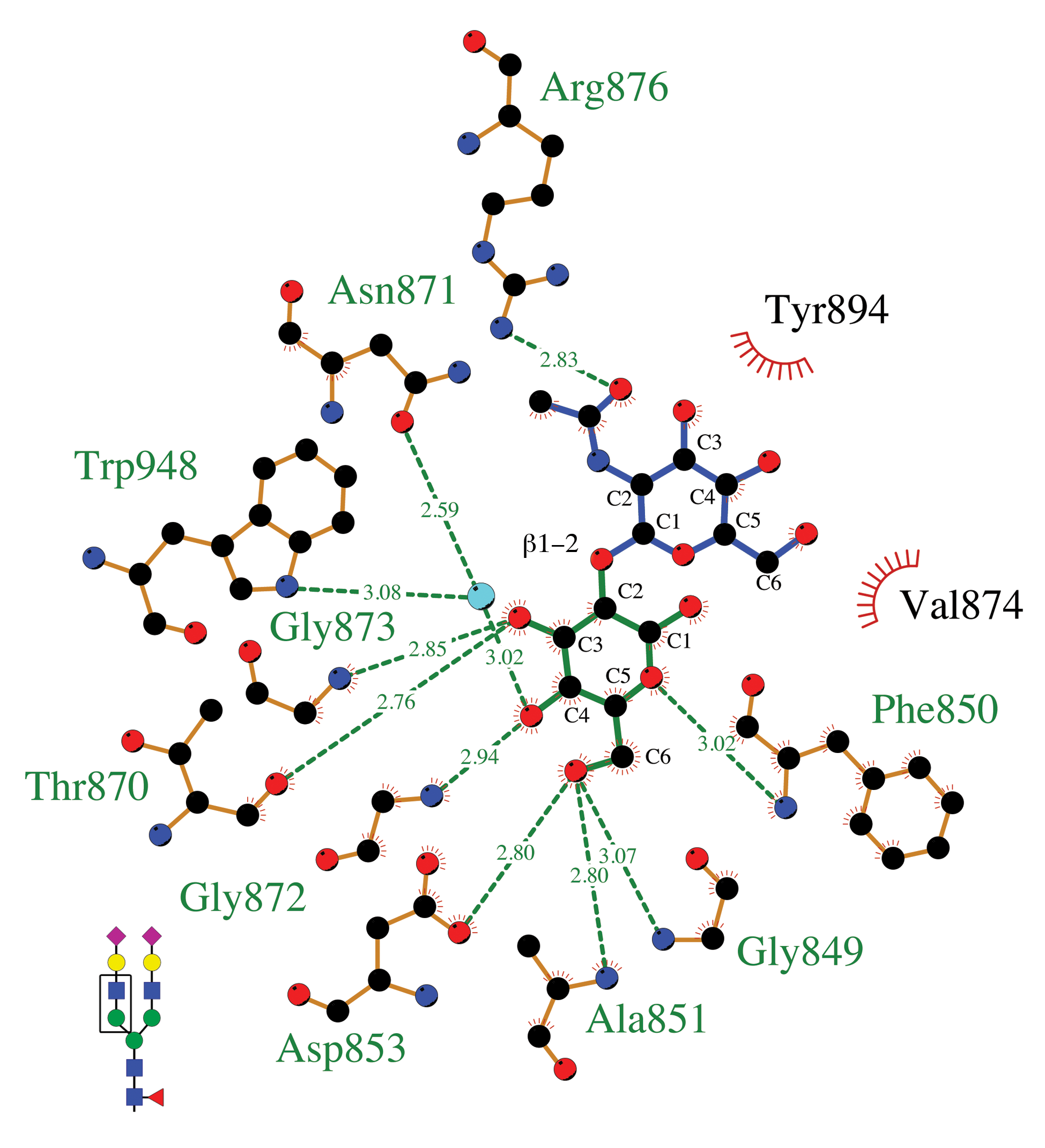

Supplement: S4 Fig — Schematic representation of hydrogen-bonding and hydrophobic contacts between RbmC2 and the GlcNAc-Man ligand. (TIF) [file ppat.1006841.s004.tif]

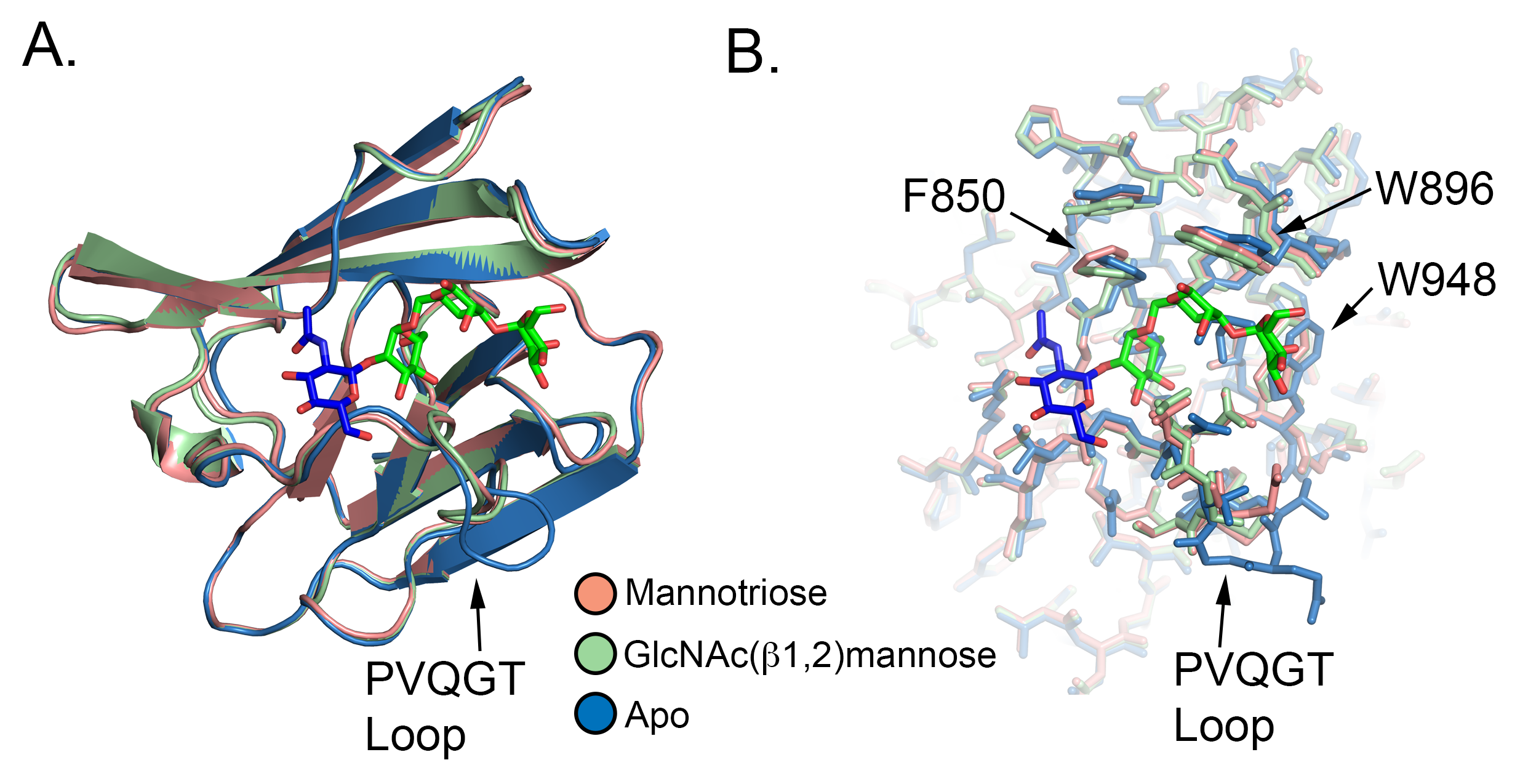

Supplement: S5 Fig — (A) Superposition between mannotriose, GlcNAc-Man, and apo structures (chain B, one of three chains in the asymmetric unit). The PVQGT loop of only one asymmetric unit domain is structured, adopting an alternative conformation to the mannotriose-bound structure loop. The composite GlcNAc-mannotriose glycan fragment is shown in a green and blue stick representation. (B) Superposition between apo (one copy), GlcNAc-Man, and mannotriose-bound RbmC2 structures illustrating slight aromatic side-chain movements that occur upon ligand binding. (TIF) [file ppat.1006841.s005.tif]

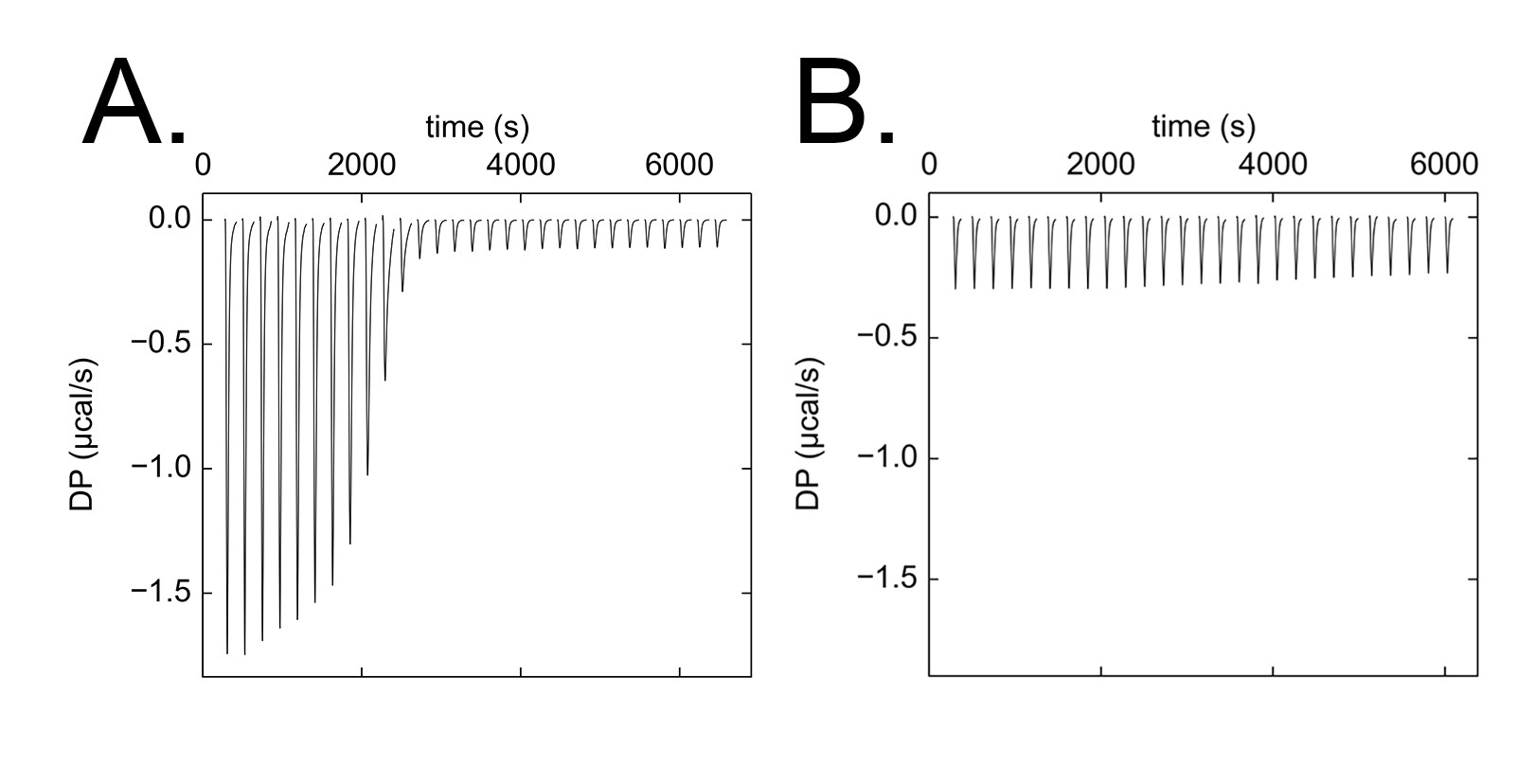

Supplement: S6 Fig — (A) ITC isotherm of asialofetuin binding to purified GFPUV-RbmC2 fusion produced by GUSSI. (B) Binding of the D853A point mutant GFPUV-RbmC2 fusion under identical conditions illustrating loss of asialofetuin affinity. (TIF) [file ppat.1006841.s006.tif]

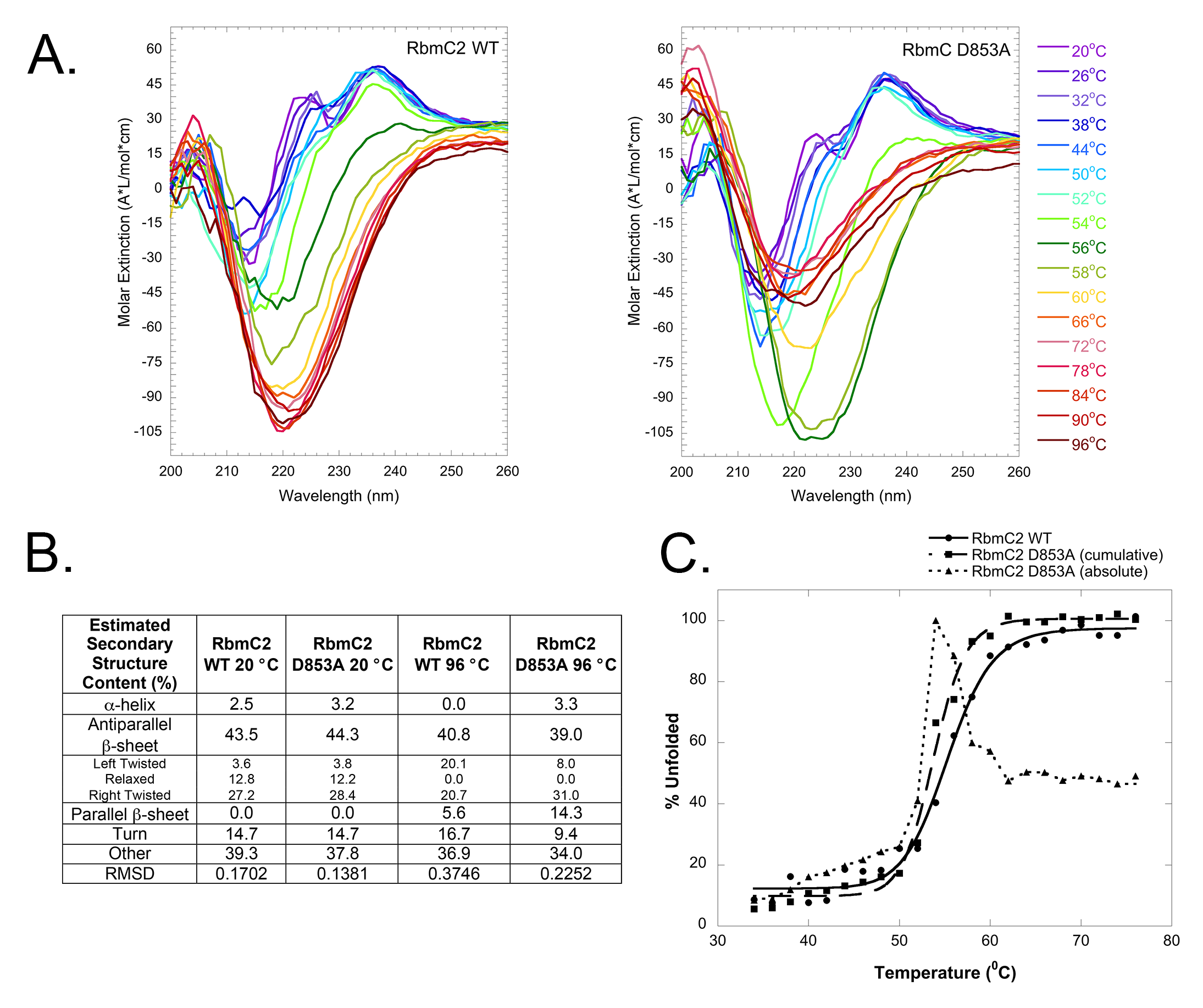

Supplement: S7 Fig — (A) Superposition of CD spectra for WT (left) and D853A (right) RbmC2 including scans for every 6°C in folded and melted ranges, every 2°C in transition range to illustrate variable unfolding pathway between 50–60°C. (B) Table illustrating percentages of secondary structure elements for each protein at 20°C and 96°C as calculated by BeStSel. Calculation of secondary structure from the RbmC2 apo crystal structure using DSSP [65] indicates 2.2% α-helix, 52.9% β-sheet, and 44.9% other. The antiparallel β-sheet category is broken down into left-twisted, relaxed, and right-twisted sheets. (C) Plot showing unfolding of RbmC2 WT and RbmC2 D853A proteins based on the CD melt data from 34 to 76°C. The mutant data is presented both as % unfolded based on the absolute shift of the CD signal and on the cumulative CD signal change from folded to unfolded states (a dip and then rise in the CD signal at 222 nm). From a sigmoidal fit of the curves, the Tm is estimated to be 55°C for WT and 54°C for the mutant. (TIF) [file ppat.1006841.s007.tif]
